# Oral of PtCuS nanoclusters mitigates acute radiation-induced intestinal injury by coordinating glutathione metabolism, macrophage repolarization, and gut microbiota

**DOI:** 10.1016/j.mtbio.2026.103403

**Published:** 2026-06-28

**Authors:** Yilin Zheng, Shengqi Yin, Yishu Zou, Zehui Zhang, Junjie Li, Jianxin Chen, Wanying Zheng, Yang Liu, Yuqin Zhang, Peiqun Yin, Yi Ding

**Affiliations:** a Department of Radiation Oncology, Nanfang Hospital, Southern Medical University, Guangzhou, Guangdong Province, 510515, China; b School of Biomedical Engineering, Research and Engineering Center of Biomedical Materials, Anhui Medical University, Hefei, 230032, China; c Guangdong Province Key Laboratory of Molecular Tumor Pathology, Guangzhou, Guangdong Province, 510515, China

**Keywords:** Acute radiation-induced intestinal injury, PtCuS nanoclusters, Glutathione metabolism, Macrophage repolarization, Gut microbiota

## Abstract

Acute radiation-induced intestinal injury (ARIII) is a common side effect of abdominopelvic radiotherapy, with severe diarrhea and hematochezia occurring in approximately 60–80% of patients. Radiation inevitably damages the adjacent intestine, generating substantial reactive oxygen species (ROS) that impair intestinal function. Nanozymes, which combine enzyme-like catalytic activities with the advantages of nanomaterials, have broad applications in biomedicine. Applying nanozymes to mitigate radiation-induced intestinal damage represents a promising therapeutic strategy. Here, we developed PtCuS nanoclusters (NCs) with favorable biocompatibility and demonstrated their efficacy in mitigating ARIII after irradiation. PtCuS NCs scavenge ROS via enzyme-mimetic activity and activate glutathione metabolism to mitigate radiation-induced cell death and inflammation. PtCuS NCs also modulate macrophage polarization, suppressing M1-like pro-inflammatory activation and promoting an M2-like reparative phenotype through both direct macrophage regulation and epithelial-protective effects. Furthermore, PtCuS NCs help restore gut microbiota composition and metabolic profiles after irradiation, providing a microbiota-associated component of intestinal protection. Importantly, PtCuS NCs alleviate ARIII without compromising the therapeutic efficacy of radiotherapy. These findings demonstrate that oral administration of PtCuS NCs may safely and effectively mitigate ARIII, highlighting their potential to improve the quality of life of patients undergoing abdominopelvic radiotherapy.

## Highlights

• PtCuS nanoclusters (NCs) mitigate acute radiation-induced intestinal injury (ARIII).
• PtCuS NCs scavenge ROS and restore glutathione metabolism.
• PtCuS NCs exert anti-inflammatory effects by reprogramming macrophage polarization.
• PtCuS NCs help restore gut microbiota composition and metabolic profiles.
• PtCuS NCs alleviate ARIII without compromising the therapeutic efficacy of radiotherapy.

## 1 Introduction

Radiotherapy is a cornerstone of the standard therapeutic regimen for abdominopelvic malignancies, particularly locally advanced rectal cancer [[1], [2], [3]]. It significantly improves local control rates and facilitates tumor downstaging [4,5]. However, despite continuous advances in radiotherapy techniques, radiation-induced injury to adjacent healthy tissues remains a major clinical challenge [6]. Ionizing radiation (IR) induces a cascade of detrimental effects, including intestinal injury, disruption of gut microbiota homeostasis, and inflammatory responses [7,8]. Clinical data indicate that 60–80% of patients experience acute radiation-induced intestinal injury (ARIII) following abdominopelvic radiotherapy, with 10–20% subsequently developing chronic manifestations [9,10]. These conditions are characterized by severe diarrhea, abdominal pain, and hematochezia, which compromise quality of life, reduce treatment tolerance, and negatively affect clinical outcomes [11]. Current management of ARIII is largely palliative and relies on symptomatic relief or surgical intervention, while standardized and effective pharmacological interventions remain limited [12]. Therefore, developing novel strategies to mitigate ARIII is essential for improving both patient quality of life and long-term survival.

Nanozymes constitute an emerging class of nanomaterials that mimic the catalytic activities of natural enzymes owing to their unique physicochemical properties [13]. Among them, catalase (CAT)-like and superoxide dismutase (SOD)-like nanozymes have emerged as promising candidates in biomedicine due to their high reactivity toward reactive oxygen species (ROS) [14]. These nanozymes exhibit favorable stability, biocompatibility, and potential for scalable production and clinical translation [15,16]. Several clinically approved nanomedicines and nanomaterials with enzyme-like properties have further encouraged the biomedical translation of nanozyme-based strategies, with applications spanning antibacterial therapy, skin barrier restoration, antitumor therapy, and protection of the hematopoietic system [[17], [18], [19]]. IR not only induces direct DNA strand breaks but also triggers water radiolysis, generating excessive ROS that exacerbate genomic instability and tissue injury [20]. Given their potent antioxidant properties, nanozymes represent a feasible and clinically promising strategy for mitigating ARIII.

Despite these promising features, monometallic nanozymes often exhibit limited catalytic activity and structural instability under physiological conditions [21]. Bimetallic systems, such as PtCu alloys, can help overcome these limitations through electronic synergy between constituent metals, enhancing catalytic performance and maintaining long-term stability [22]. Beyond composition, the structural form of the nanozyme is also critical. Compared with crystalline nanoparticles (NPs), amorphous nanoclusters (NCs) possess smaller size, abundant unsaturated surface sites, and irregular atomic arrangements, which collectively improve ROS scavenging efficiency and biostability [23,24]. Furthermore, heteroatom coordination, such as sulfur coordination, can modulate the local electronic structure and enhance catalytic performance [25]. The combination of bimetallic synergy, amorphous nanocluster architecture, and sulfur-mediated electronic modulation provides a rational design strategy for highly active and biocompatible nanozymes. Accordingly, PtCu nanoparticles (NPs) and PtCuS nanoclusters (NCs) were synthesized and systematically evaluated for their efficacy in mitigating acute radiation-induced intestinal injury.

## 2 Materials and methods

### 2.1 Synthesis of PtCu NPs and PtCuS NCs

In a typical synthesis of PtCu alloy nanoparticles, 20 mg Pt(acac)_2_, 13 mg Cu(acac)_2_, 100 mg polyvinylpyrrolidone, 50 mg KI were dissolved in 10 mL formamide. The resulting homogeneous blue solution was transferred to a glass pressure vessel. The vessel was then heated at 150 °C for 3.0 h before it was cooled to room temperature. The dark products were precipitated by acetone, separated via centrifugation and further purified with an ethanol–acetone mixture.

In addition to PtCu NPs, we synthesized PtCuS alloy nanoclusters containing an additional 50 mg of glutathione (GSH). The resulting homogeneous light yellow solution was transferred to a glass pressure vessel. The vessel was then heated at 150 °C for 3.0 h before it was cooled to room temperature. The dark brown products were precipitated by acetone, separated via centrifugation, and further purified with an ethanol–acetone mixture. Based on the precursor feeding amounts (20 mg of Pt(acac)_2_ and 13 mg of Cu(acac)_2_), approximately 16 mg of solid PtCuS NCs was obtained, corresponding to a practical yield of approximately 64%. X-ray diffraction (XRD) analysis confirmed high phase purity of PtCuS NCs, with no detectable metallic Pt, Cu, or secondary copper sulfide phases (Fig. S1A). The synthesis is conducted under mild temperature and atmospheric conditions with commercially available and inexpensive precursors, supporting excellent scalability. A 10-fold scale-up of the reaction produced PtCuS NCs with unchanged phase structure, morphology, or particle size (Fig. S1A and B).

### 2.2 Cell culture and cell construction

The IEC-6 and NCM460 cell lines were purchased from Shanghai Cell Bank. The MC38, CT26, DLD-1 and HCT15 cell lines were generously provided by the department of Pathology of Nanfang Hospital. These cells were cultured in Dulbecco's Modified Eagle's Medium (DMEM; Gibco, USA) supplemented with 10% fetal bovine serum (FBS; Gibco, USA) and maintained at 37 °C in a humidified atmosphere containing 5% CO_2_ [26]. Buthionine sulfoximine (BSO) was purchased from Selleck and dissolved in distilled water. After pretreatment with BSO for 24 h, cells were subjected to subsequent experimental procedures.

### 2.3 Mouse model

The mouse model utilized 5–6 week-old male C57BL/6 mice, which were procured from the Guangdong Medical Laboratory Animal Center. All experiments involving animals were conducted according to the ethical policies and procedures approved by the Animal Ethics Committee of Guangdong Medical Laboratory Animal Center (Approval Nos. C202304-4 and D202511-12).The mice were housed under specific pathogen-free conditions with ad libitum access to food and water. Following a 1-week acclimatization period, the mice were randomly assigned to the various experimental groups.

The ARIII model was established by delivering a single 12 Gy dose of abdominopelvic irradiation using a linear accelerator (Varian Clinac 23EX Linear Accelerator, USA). PtCu NPs and PtCuS NCs were administered by oral gavage at a dose of 5 mg/kg for five consecutive days, after which intestinal injury was evaluated. For chronic injury assessment, we lowered the irradiation dose to 10 Gy and the 2-month time point after initial gavage was used as the observation window, based on the progression of the acute radiation-induced intestinal injury model. For the rescue study, mice were given intraperitoneal injections of BSO (5 mmol/kg; dissolved in distilled water, 10 mL/kg) for five consecutive days [27]. For benchmarking therapeutic effects, amifostine (200 mg/kg; 30 min before irradiation) was included as a positive control and administered by intraperitoneal injection [28]. For the macrophage depletion study, mice were treated with intraperitoneal injections of clodronate liposomes (100 μL per 10 g body weight) suspended in phosphate-buffered saline (PBS). The injections were given 2 d before and 2 d after irradiation to achieve systemic depletion of macrophages [29]. For the orthotopic colorectal cancer model, MC38 cells suspension (1 × 10^6^ cells in PBS/Matrigel [1:1]) was injected into the ssubserosal layer of the cecal wall of the male C57BL/6 mice (5–6 weeks old), mice were randomly divided into four groups for further treatment. A single 10 Gy dose of abdominopelvic irradiation was performed on days 14 after implantation, followed by oral administration of the PtCuS NCs for five consecutive days. Mice were subsequently euthanized, and tissues were collected for further analysis.

### 2.4 AAV-mediated *GCLC* knockdown in mice

To induce glutamate cysteine ligase catalytic subunit (*GCLC*) knockdown and evaluate its effect on intestinal radiation injury, 5-week-old mice were administered adeno-associated virus (AAV) vectors carrying *GCLC*-targeting shRNA. The AAV vectors were delivered via intraperitoneal injection at a dose of 5 × 10^11^ viral genomes per mouse. Knockdown efficiency was assessed 3 weeks after injection by measuring GCLC expression in intestinal tissue using quantitative polymerase chain reaction (qPCR) and western blotting analysis.

### 2.5 IR treatment and colony formation assays

Intestinal cells and tumor cells were exposed to irradiation doses from 0 to 8 Gy using a linear accelerator (Varian Clinac 23EX Linear Accelerator, USA). For the colony formation assays, cells were seeded in six-well plates in replicate sets. The following day, cells were treated with PtCu NPs or PtCuS NCs and irradiated at the indicated doses (0–8 Gy). After irradiation, the cells were maintained in fresh culture medium, which was changed every 3 days, and incubated for 2–3 weeks to allow colony formation. When the clone clusters were observed, the medium was discarded; the cells were washed with PBS and fixed with ice-cold methanol for 30 min, then stained with 0.5% crystal violet for 1 h at room temperature. After thorough washing with distilled water, visible colonies (>50 cells per colony) were counted using ImageJ software (National Institutes of Health, Bethesda, MD, USA). The surviving fraction at each radiation dose was calculated using the established formula: [Surviving Fraction = (number of surviving colonies in dose D)/(number of cells seeded for dose D) × (average colonies arising from the nonirradiated cells (0 Gy)/number of nonirradiated cells seeded)]. Dose–survival curve was then plotted using GraphPad Prism 9.0 software, and survival curves were plotted according to the single-hit multi-target model SF = 1 − (1 − e^−D/D0)^N, where SF is the surviving fraction, D is the IR dose and N is the extrapolation number [30].

### 2.6 Histological processing and hematoxylin and eosin (H&E) staining

Mouse intestinal tissue was isolated, washed with ice-cold PBS and immediately fixed in 10% neutral–buffered formalin for 24–48 h at 4 °C. The tissue was dehydrated by exposure to a graded ethanol series (70%, 80%, 90%, 95%, and 100%) and cleared with xylene. Then tissues were embedded in paraffin wax, cut into sections (4 μm thickness), mounted on slides, and stained with H&E according to the manufacturer's instructions (Solarbio, Beijing, China). Staining of pathological injury was independently evaluated by two senior pathologists who were blinded to group allocation. The histopathological scoring system was based on the following criteria: 0 (normal): no pathological alterations; 1 (mild): focal separation of submucosa and/or lamina propria; 2 (moderate): evident submucosal/lamina propria separation and presence of submucosal and muscular layer edema; 3 (severe): extensive architectural disruption, marked submucosal edema and villous epithelial detachment; and 4 (necrosis): complete villous destruction and transmural necrosis [31]. Each group was quantified with at least five different views.

### 2.7 Alcian blue-periodic acid schiff staining and immunohistochemistry

Goblet cells were identified using Alcian blue-periodic acid–Schiff (AB-PAS) staining with a commercial kit (AB-PAS Stain Kit; Solarbio, Beijing, China) according to the manufacturer's protocol. Stained sections were imaged using a digital microscope (DP22, Olympus) with consistent illumination settings. Goblet cell staining was independently evaluated by two senior pathologists who were blinded to group allocation. Goblet cells were quantified by counting all positively stained cells showing characteristic magenta/blue cytoplasmic staining in crypts within three randomly selected fields per mouse.

Immunohistochemistry (IHC) staining of intestinal and tumor tissue sections was performed according to the standard protocols. Primary antibodies were γH2AX-Ser139 (1:200, #9718, Cell Signaling Technology, USA), LGR5 (1:150, OTI2A2, Invitrogen, USA), PCNA (1: 2000, A12427, Abclonal, China), CD8a (1: 2000, ab217344, Abcam, USA) and CD206 (1: 800, 2459, Cell Signaling Technology, USA). Staining was independently evaluated by two senior pathologists who were blinded to group allocation. Immunoreactivity was quantified as the percentage of positive area relative to the total tissue area using ImageJ software. For each mouse or tissue sample, at least three randomly selected fields were analyzed.

### 2.8 Immunofluorescence (IF) staining

For *in vitro* IF staining, intestinal epithelial cells were seeded in confocal dishes for 24–48 h, and then exposed to 8 Gy irradiation. After 48–72 h, cells were fixed using 4% paraformaldehyde for 1 h, permeabilized using 0.3% Triton X-100 for 5 min, blocked with goat serum for 30 min, incubated with the primary antibody (γH2AX-Ser139, 1:200, #9718, Cell Signaling Technology, USA) overnight and secondary antibody (Alexa-488-conjugated goat anti-Rabbits secondary antibody, 1: 200, bs-0295G-BF488, Bioss, China) for 1 h and then stained for DNA with 4′,6-diamidino-2-phenylindole (DAPI; 1 μg/mL) for 3 min. Images were observed with a confocal laser scanning microscope (Olympus FV2000). The γH2AX positive foci of each cell were quantified using ImageJ software. Each group was quantified using at least ten different cells.

For *in vivo* study, tissues were dehydrated and embedded in paraffin wax, sectioned (4 μm thickness), and mounting on slides. Tissues were deparaffinized, rehydrated, and antigen-unmasked following standard protocols. Then tissues were permeabilized with 0.3% Triton X-100 in PBS for 15 min, blocked with goat serum for 30 min, incubated with primary antibody overnight and secondary antibody for 1 h, and then stained with DAPI (1 μg/mL) for 5 min. Primary antibodies were ZO-1 (1: 200, 61-7300, Invitrogen, USA), Claudin3 (1:100, 34-1700, Invitrogen, USA), MPO (1: 400, 22225-1-AP, Proteintech, China), F4/80 (1:50, ab300421, Abcam, USA), CD206 (1: 800, #245958, Cell Signaling Technology, USA) and NOS2 (1: 500, ab178945, Abcam, USA). Secondary antibodies were Alexa-488-conjugated goat anti-rabbit antibody (1: 200, bs-0295G-BF488, Bioss, China) and Alexa-555-conjugated goat anti-mouse antibody (1: 200, bs-0368G-BF555, Bioss, China). Staining was independently evaluated by two senior pathologists who were blinded to group allocation. The mean fluorescence intensity and fluorescence-positive area were quantified using ImageJ software. For each mouse or tissue sample, at least three randomly selected fields were analyzed.

### 2.9 TUNEL staining

Apoptotic cells were detected using commercial kit and followed the manufacturer's instructions (Beyotime Biotechnology, China). Briefly, paraffin–embedded sections were deparaffinized, permeabilized with Proteinase K (20 μg/mL, 20 min), and incubated with TUNEL reaction mixture (60 min, 37 °C). Nuclei were incubated with DAPI for 5 min. Images were observed with a confocal laser scanning microscope (Olympus FV2000). Staining was independently evaluated by two senior pathologists who were blinded to group allocation. The fluorescence area were quantified by ImageJ software. Each group was quantified using at least five random fields.

### 2.10 Western blotting analysis

Cells or tissues were lysed with RIPA buffer (Beyotime Biotechnology) supplemented with protease and phosphatase inhibitors (CWBIO, China). Total proteins were separated by 10% or 12% SDS-PAGE and transferred onto PVDF membranes. The blots were blocked and then incubated with primary antibodies overnight followed by incubation with HRP-conjugated secondary antibodies for 1 h at 4 °C. [26]. Proteins were detected using enhanced chemiluminescence (FD Bio-pico ECL, China). Primary antibodies were as follows β-Actin (66009-1-Ig, Proteintech, China), ZO-1 (61-7300, Invitrogen, USA), Claudin3 (34-1700, Invitrogen, USA), Bcl-XL (10783-1-AP, Proteintech, China), Caspase 3/p17/p19 (19677-1-AP, Proteintech. China), GPX4 (67763-1-Ig, Proteintech, China), Slc7a11 (T57046, Abmart, China), p-MLKL (ab196436, Abcam, USA), and p-RIPK1 (66854-1-Ig, Proteintech, China). Secondary antibodies were HRP-conjugated goat anti-rabbit IgG(H + L) (SA00001-2, Proteintech, China) and HRP-conjugated goat anti-mouse IgG(H + L) (SA00001-1, Proteintech, China).

### 2.11 Quantitative real-time PCR (qPCR)

Total RNA was isolated from cells or tissues using TRIzol reagent (AG, China) following the manufacturer's protocol. cDNA was synthesized using Evo M-MLV RT Master Mix (AG). Quantitative PCR was carried out using SYBR Green Premix Pro Taq HS qPCR Kit (Rox Plus) (AG) on a LightCycler 96 system (Roche). Gene expression levels were normalized to β-Actin, and relative quantification was calculated using the 2^−ΔΔCt method.

### 2.12 Cell proliferation assay

Cell proliferation was evaluated using a 5-ethynyl-2′-deoxyuridine (EdU) assay kit (Beyotime Biotechnology). Cells were seeded in 24-well plates (3000 cells/well) and cultured for 72 h following 8 Gy irradiation. The proliferating cells were labeled by incubating with 100 μM EdU for 2 h at 37 °C. Cells were then fixed with 4% formaldehyde (30 min), permeabilized with 0.3% Triton X-100 (10 min), and stained according to the manufacturer's protocol. Nuclei were counterstained with Hoechst 33342. EdU-positive cells were imaged using fluorescence microscopy (Olympus FV1000) and quantified using ImageJ software. Image quantification was performed by two independent investigators who were blinded to group allocation. At least five randomly selected fields were analyzed per group, and proliferation rates were calculated as the percentage of EdU-positive cells relative to total nuclei.

### 2.13 Biodistribution of PtCu nanozymes

Cy7 powder was dissolved in PBS and mixed with PtCu NPs or PtCuS NCs by gentle shaking for 24 h at 4 °C to construct PtCu NPs@Cy7 and PtCuS NCs@Cy7. Six-week-old male C57BL/6 mice were fasted for 12 h and then gavaged with PtCu NPs@Cy7 or PtCuS NCs@Cy7. Biodistribution in the gastrointestinal tract, heart, liver, spleen, lung, and kidney was assessed at different time points (1 h, 4 h, 8 h, and 12 h). After gavage, fluorescence imaging was performed, and the mean fluorescence intensity of regions of interest (ROIs) was analyzed using Bruker MI SE software (Bruker, Billerica, MA, USA). The fluorescence intensity was expressed as 10^4^ p/s/mm^2^.

### 2.14 Enzyme-linked immunosorbent assay (ELISA)

Blood samples were collected from mice and centrifuged at 500 × g for 8 min at 4 °C to obtain serum. For intestinal tissue analysis, samples (0.5 g) were homogenized in 1000 μL of ice-cold PBS and centrifuged at 5000 × g for 10 min at 4 °C. The supernatants from both serum and tissue homogenates were analyzed for IL-6, TNFα, and IL-1β concentrations using commercial ELISA kits (Neobioscience, China) following the manufacturer's protocols. Absorbance was measured at 450 nm, and cytokine levels were calculated from standard curves.

### 2.15 Flow cytometry (FCM)

The production of ROS by irradiated cells and tissues was measured using a ROS assay kit (YEASEN, Shanghai, China) according to the manufacturer's protocol.

For the apoptosis assay, cells were pretreated according to the experimental design, and then harvested and stained using an Annexin V-FITC/DAPI Apoptosis Detection Kit (KeyGEN BioTECH, China) according to the manufacturer’ s protocol. Apoptotic cells were quantified by flow cytometry (BD FACSCanto II), and the data were analyzed using FlowJo software. Early apoptotic (Annexin V^+^/DAPI^−^) and late apoptotic/necrotic (Annexin V^+^/DAPI^+^) populations were distinguished.

For the investigation of immune cell phenotype, intestinal tissues were collected and washed with PBS. The tissue was cut into pieces and incubated with PBS containing DNase I and collagenase IV with horizontal shaking (220 rpm, 37 °C, 30 min). The resulting cell suspension was filtered through a 70 μm nylon mesh and centrifuged (300 × g, 5 min, 4 °C). The cells were resuspended in Hank's balanced salt solution buffer (HBSS) and washed twice. Subsequently, cells were incubated with antibodies according to the experimental design. The surface antibodies included FITC anti-mouse CD45 antibody (S18009F, BioLegend, San Diego, CA, USA), PE anti-mouse/human CD11b antibody (M1/70, BioLegend), PE/Cyanine 7 anti-mouse F4/80 antibody (BM8, BioLegend), APC anti-mouse Ly-6G antibody (1A8, BioLegend), Brilliant Violet 421™ anti-mouse CD206 (MMR) antibody (C068C2, BioLegend) and Brilliant Violet 650™ anti-mouse CD86 antibody (GL-1, BioLegend).

For the investigation of tumor immune cell phenotypes, tumor tissues were treated following the same procedure. The surface antibodies including FITC anti-mouse CD45 antibody (S18009F, BioLegend), APC anti-mouse CD3 antibody (17A2, BioLegend) and PE anti-mouse CD8a antibody (53-6.7, BioLegend). Then cells were fixed and permeabilized for intracellular staining according to the manufacturer's instructions (BD 334714 Fixation/Permeabilization kit, BD Pharmingen, San Diego, CA, USA). The intracellular antibody includes Brilliant Violet 421™ anti-human/mouse granzyme B recombinant antibody (QA16A02, BioLegend). Data were acquired on a BD FACSCanto II and analyzed using FlowJo software (BD Biosciences, San Diego, CA, USA).

### 2.16 FITC–dextran for intestinal integrity

Six-week-old male C57BL/6 mice were fasted for 12 h to standardize gastrointestinal conditions. FITC–dextran (3-5 kDa, Sigma-Aldrich) was dissolved in PBS. FITC–dextran was administered by oral gavage at a dose of 20 mg per 20 g body weight in 0.2 mL PBS [32]. Blood samples were collected and serum samples were isolated by centrifugation at 500 × g for 5 min at 4 °C. Then the serum samples were diluted 1:10 in PBS, and the fluorescence intensity was measured at an excitation wavelength of 485 nm and an emission wavelength of 535 nm.

### 2.17 Isolation of bone marrow-derived macrophages (BMDM)

After six-week-old male C57BL/6 mice were euthanized, bilateral femurs and tibiae were aseptically dissected in a biological safety cabinet, with care to preserve bone integrity. Bone marrow cells were flushed from the marrow cavities under sterile conditions to generate a cell suspension, which was filtered through a 70 μm strainer to obtain a single-cell suspension. Red blood cells were lysed in red blood cell lysis buffer for 5 min at room temperature. The lysis reaction was terminated by adding an equal volume of complete medium, followed by centrifugation at 500 × g for 5 min at 4 °C. The resulting cell pellet was resuspended in complete DMEM medium supplemented with 10% FBS, 1% penicillin–streptomycin, and 20 ng/mL macrophage colony-stimulating factor (M-CSF), and cultured for 5–7 days until maturation. Mature BMDMs were then used for subsequent induction and experiments.

### 2.18 Small intestinal organoid culture and treatment

Small intestinal crypts were isolated from 5–6-week-old male C57BL/6 mice under sterile conditions. Briefly, the small intestine was excised, opened longitudinally, and washed repeatedly with cold PBS. The tissue was cut into small pieces and incubated in EDTA-containing dissociation buffer to release intestinal crypts. After mechanical dissociation, the crypt suspension was filtered and centrifuged. The crypt pellet was mixed with cold Matrigel and plated as droplets in culture plates. After Matrigel polymerization at 37 °C, intestinal organoid culture medium was added, and the medium was replaced every 2–3 days.

For functional assays, established organoids were assigned to the indicated treatment groups. PtCuS NCs were added to the culture medium before irradiation. In the BSO rescue experiment, organoids were pretreated with BSO to inhibit GSH synthesis before PtCuS NC treatment and irradiation. Organoids were exposed to 8 Gy irradiation and further cultured under the corresponding treatment conditions. Bright-field images were captured at the indicated time points using an inverted microscope. Organoid morphology and regenerative growth were assessed by quantifying organoid diameter, bud number, and bud length with ImageJ software. At least ten organoids were analyzed per group unless otherwise indicated.

### 2.19 RNA sequencing (RNA-seq)

Intestinal tissues were collected from C57BL/6 mice subjected to 12 Gy irradiation with or without PtCuS NC treatment. At 5 days after irradiation, total RNA was extracted using TRIzol reagent (Ambion/Invitrogen, USA) and RNA-seq libraries were constructed. The quality of the RNA-seq libraries was analyzed using the NGS 3K/Caliper/DNF-915 (Perkin Elmer, USA). Library sequencing was performed on a NovaSeq sequencing platform (Illumina, USA) by Novogene Corp., China.

### 2.20 16S gene sequencing and analysis

Total microbial genomic DNA was extracted from fecal samples using the FastPure Stool DNA Isolation Kit (MJYH, Shanghai, China) according to the manufacturer's instructions. The quality and concentration of the DNA were determined by 1.0% agarose gel electrophoresis and a NanoDrop ND-2000 spectrophotometer (Thermo Scientific Inc., USA), and samples were stored at −80 °C until use.

Raw reads were quality-filtered using fastp (https://github.com/OpenGene/fastp, version 0.19.6) to remove adapters and low-quality bases [33]. Paired-end reads were merged using FLASH (version 1.2.11) [34]. Bioinformatic analysis of the gut microbiota was performed on the Majorbio Cloud platform (https://cloud.majorbio.com). Based on operational taxonomic units (OTU) data, Mothur v1.30.2 was employed to compute rarefaction curves, alpha and beta diversity indices. The linear discriminant analysis effect size (LEfSe) analysis was performed to identify differentially abundant bacterial taxa from phylum to genus among the different groups, with an LDA score >2 and P < 0.05 considered significant [35].

### 2.21 Non-targeted metabolomics analysis

Fecal metabolomics data were analyzed using the Majorbio Cloud Platform (cloud.majorbio.com) [36]. The LC–MS/MS analysis of fecal samples was conducted on a Thermo UHPLC-Q Exactive HF-X system equipped with an ACQUITY HSS T3 column (100 mm × 2.1 mm i.d., 1.8 μm; Waters, USA) at Majorbio Bio-Pharm Technology Co. Ltd. (Shanghai, China). The mobile phases consisted of 0.1% formic acid in water:acetonitrile (95:5, v/v) (solvent A) and 0.1% formic acid in acetonitrile:isopropanol:water (47.5:47.5:5, v/v/v) (solvent B). The flow rate was 0.40 mL/min and the column temperature was 40 °C. The injection volume was 3 μL. The mass spectrometric data were collected using a Thermo UHPLC-Q Exactive HF-X mass spectrometer equipped with an electrospray ionization source operating in positive and negative ion modes. The UHPLC–MS raw data were converted into a common format by Progenesis QI software (Waters, Milford, USA) through baseline filtering, peak identification, peak integration, retention time correction, and peak alignment. The data matrix containing sample names, *m*/*z* values, retention times, and peak intensities was then exported for further analysis. Metabolites were identified using HMDB (http://www.hmdb.ca/), Metlin (https://metlin.scripps.edu/), and the in-house Majorbio database of Majorbio Biotechnology Co., Ltd. (Shanghai, China).

### 2.22 Statistical analysis

Data were analyzed using Prism 9.0 (GraphPad, San Diego, CA, USA) and are presented as the mean ± standard deviation (SD). *In vitro* experiments were independently repeated at least three times unless otherwise indicated. For animal experiments, each individual mouse was considered one biological replicate, and the exact number of mice per group is indicated in the corresponding figure legends. When technical replicates were included, their mean value was used for statistical analysis. Histological and immunostaining evaluations were performed in a blinded manner by two senior pathologists who were unaware of the treatment groups. For two-group comparisons, an unpaired two-tailed Student's t-test was used when the equal variance assumption was met, whereas Welch's *t*-test was applied for comparisons with unequal variances. For comparisons among more than two groups, one-way analysis of variance (ANOVA) followed by Tukey's multiple-comparison test was used. For experiments involving two independent variables, two-way ANOVA followed by Tukey's multiple-comparison test was applied. Clonogenic survival curves were fitted by nonlinear regression and compared using the extra sum-of-squares F test. Survival related to ARIII was assessed using Kaplan–Meier curves and compared using the log-rank (Mantel–Cox) test. Body weight changes over time were analyzed using a mixed-effects model with repeated measures. Statistical significance was defined as *p* < 0.05; significance levels are indicated as ∗*p* < 0.05, ∗∗*p* < 0.01, ∗∗∗*p* < 0.001, and ∗∗∗∗*p* < 0.0001.

## 3 Results

### 3.1 Synthesis and characterization of PtCu NPs and PtCuS NCs

**Fig. 1:**
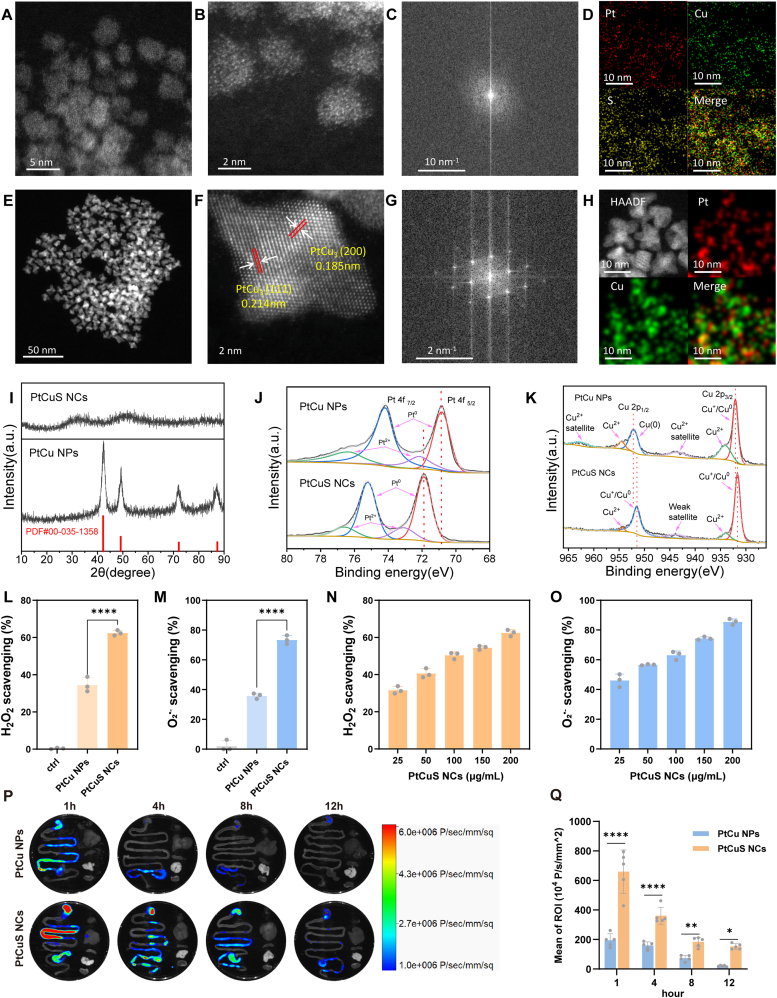
Characterization of PtCuS NCs and PtCu NPs. (A, E) Representative image of HAADF-STEM image of PtCuS NCs and PtCu NPs, respectively. (B, F) Aberration corrected HAADF-STEM with sub-angstrom resolution image of PtCuS NCs and PtCu NPs, respectively. (C, G) The FFT pattern of PtCuS NCs and PtCu NPs, respectively. (D, H) HAADF-STEM image and the corresponding EDX elemental mappings. (I) XRD patterns of PtCuS NCs and PtCu NPs. (J, K) XPS High-resolution spectra of Pt 4f and Cu 2p of PtCuS NCs and PtCu NPs. (L, M) H_2_O_2_ scavenging efficacy and O_2_^•–^ scavenging efficacy of PtCuS NCs and PtCu NPs in 200 μg/mL concentration (*n* = 3). (N, O) H_2_O_2_ scavenging efficacy and O_2_^•–^ scavenging efficacy of PtCuS NCs (*n* = 3). (P, Q) Representative distribution and statistics of fluorescence in the vital organs and intestine (*n* = 4). Data are presented as mean ± SD. Each dot in panel Q represents one biological replicate from an individual mouse. Statistical significance was determined by one-way ANOVA followed by Tukey's multiple-comparison test for panel L, M and Q. ∗*p* < 0.05, ∗∗*p* < 0.01, ∗∗∗*p* < 0.001, ∗∗∗∗*p* < 0.0001.

PtCu NPs were synthesized using a one-pot oil bath method and modified with polyvinylpyrrolidone (PVP) as the surface capping agent. Ultrasmall PtCuS NCs were further synthesized by adding glutathione. The synthesis yielded high-purity, uniform PtCuS NCs with good scalability (Fig. S1A and B). High-angle annular dark-field scanning aberration-corrected scanning transmission electron microscopy (HAADF-STEM) with atomic spatial resolution was used to measure the atomic distribution and morphology. The calculated particle size of PtCuS NCs was determined to be approximately 3 nm (Fig. 1A). The HAADF-STEM image (Fig. 1B) showed no discernible lattice fringes or ordered atomic arrangements within the NCs, confirming their amorphous nature, as further supported by the diffuse pattern in the corresponding fast Fourier transform (FFT) image (Fig. 1C). Due to their lower atomic numbers, Cu and S are less prominent in HAADF imaging compared to Pt, making them difficult to resolve as distinct bright spots. Energy-dispersive X-ray (EDX) elemental mapping verified the successful synthesis of PtCuS NCs, revealing uniform distribution of Pt, Cu and S throughout the PtCuS NCs (Fig. 1D).

In contrast, the HAADF‐STEM images showed that the average size of dendritic PtCu NPs was approximately 10 nm (Fig. 1E). The ordered fringe pattern confirmed their good crystallinity, with interplanar spacing of 0.214 and 0.185 nm corresponding to the (111) and (200) facets of PtCu_3_, respectively (Fig. 1F). The corresponding FFT images of the PtCu NPs corroborated the crystalline structure (Fig. 1G). Elemental mapping further demonstrated that PtCu NPs were successfully synthesized (Fig. 1H).

The diffraction peaks of the PtCu NPs at 42.2, 49.2, 72.1, and 87.3° in the XRD pattern were indexed to the standard peaks of the PtCu_3_ diffraction file (JCPDS No. 35-1358) (Fig. 1I). Notably, PtCuS NCs showed no distinct diffraction peaks, which is consistent with their ultrasmall particle size and amorphous nature, a feature that may favor enhanced catalytic reactivity. EDX analysis and quantitation detection using inductively coupled plasma optical emission spectrometry (ICP-OES) demonstrated the coexistence of Pt and Cu elements with the Pt/Cu molar ratios of approximately 1:1.76 for PtCuS NCs and 1:3 for PtCu NPs.

X-ray photoelectron spectroscopy (XPS) was used to characterize the surface electronic states and chemical composition of the PtCuS NCs and PtCu NPs. Notably, compared with the PtCu NPs, the Pt 4f_7/2_ peak of PtCuS NCs shifted toward higher binding energy, with a shift of ∼1.0 eV (Fig. 1J and K). The Pt 4f_7/2_ peaks at 71.9 and 73.1 eV were assigned to the Pt (0) and Pt (+2) oxidation states of PtCuS NCs, respectively. This electronic modulation originates from sulfur doping, in which electronegative S induces electron transfer from metal sites to S atoms, decreasing the electron density on Pt and elevating its binding energy. In contrast, the Cu 2p peaks of PtCuS NCs shifted to lower binding energy relative to those of PtCu NPs. Such opposite shift trends of Pt and Cu suggest evident electronic interactions among Pt, Cu and S, which may effectively regulate surface electronic properties.

The hydrodynamic sizes of PtCuS NCs and PtCu NPs were determined using dynamic light scattering (DLS), and were approximately 5.21 and 11.03 nm, respectively (Fig. S1C). Finally, to evaluate the stability of PtCuS NCs under gastrointestinal conditions, the zeta potentials of PtCuS NCs at different pH values(7.0 and 1.5) were examined separately. Gastric acid was simulated using a dilute HCl solution (pH = 1.5) (Fig. S1D). Owing to the abundance of H^+^, the zeta potential of PtCuS NCs in HCl solutions decreased compared with that in water. Furthermore, the activities of CAT and total SOD were determined. In contrast to PtCu NPs, the PtCuS NCs demonstrated enhanced enzymatic activity at the same concentration (200 μg/mL) (Fig. 1L and M). Besides that, PtCuS NCs also showed concentration-dependent CAT- and SOD-like activities across the tested concentration range (25-200 μg/mL) (Fig. 1N and O). To examine the bimetallic synergy enhanced by sulfur doping, steady-state kinetic assays were performed. For CAT-like activity, the amorphous PtCuS NCs exhibited a lower Michaelis–Menten constant (*K*_*m*_) and a higher maximum reaction velocity (*V*_max_) than their crystalline PtCu counterparts (Fig. S2), indicating stronger substrate affinity and faster catalytic turnover. Furthermore, the kinetic parameters of PtCuS NCs surpassed those of most reported Pt/Cu-based nanozymes (Table S1). Given the high levels of reactive oxygen species (ROS) and positively charged proteins in inflamed intestinal sites, the negatively charged PtCuS NCs are expected to efficiently target inflammatory lesions and exert potent ROS-scavenging effects following oral administration.

### 3.2 Biocompatibility of PtCu NPs and PtCuS NCs

Biocompatibility is a critical prerequisite for the biomedical utilization of nanozymes [37]. *In vitro* colony formation assays were performed using rat intestinal epithelial cells (IEC-6) and human colon epithelial cells (NCM460). Both PtCu NPs and PtCuS NCs exhibited negligible cytotoxicity over a wide concentration range, with cell survival rates consistently exceeding 90% (Fig. S3A-D). *In vivo*, six-week-old male C57BL/6 mice were orally administered increasing doses of PtCu NPs and PtCuS NCs for seven consecutive days and sacrificed on day 31 (Fig. S3E). Even at the highest dose tested (20 mg/kg), no significant changes in body weight or colorectum length were observed (Fig. S3F-H). Serum liver and kidney function indicators, including AST, ALT, CREA and UREA, showed no evident organ dysfunction after oral administration of PtCu NPs or PtCuS NCs (Fig. S3I). In addition, H&E staining of major organs, including the heart, liver, spleen, kidney, and intestine, revealed no obvious pathological abnormalities or nanozyme accumulation compared with the PBS-treated group (Fig. S3J and K). To evaluate the intestinal retention of the nanozymes after oral administration, Cy7-labeled PtCu NPs and PtCuS NCs were administered to mice by gavage, and their fluorescence distributions and intensities were monitored over time. Both nanozymes reached the small intestine within 1 h after administration, whereas PtCuS NCs exhibited stronger and more persistent intestinal fluorescence than PtCu NPs at the examined time points. Notably, PtCuS NCs could still be detected in the intestinal tract 12 h after administration. *Ex vivo* fluorescence imaging of major organs, including the heart, liver, spleen, lung, and kidney, showed no obvious fluorescence signal under the current imaging conditions (Fig. 1P and Q). To further support these observations, inductively coupled plasma mass spectrometry (ICP-MS) was used to quantify the Pt level in the whole intestinal tract, from the duodenum to the rectum, at 1, 4, 8, and 12 h after oral administration. Consistent with the fluorescence results, PtCuS NCs showed higher retention than PtCu NPs at all time points, supporting the enhanced intestinal residence of PtCuS NCs (Table S2). These results support the enhanced short-term intestinal residence of PtCuS NCs after oral administration, which may contribute to sustained local ROS scavenging at injured intestinal sites. Taken together with the biosafety evaluations, these results support the favorable short-term *in vivo* biosafety and biocompatibility of PtCu NPs and PtCuS NCs under the tested conditions.

### 3.3 PtCuS NCs mitigate radiation-induced intestinal injury *in vitro*

**Fig. 2:**
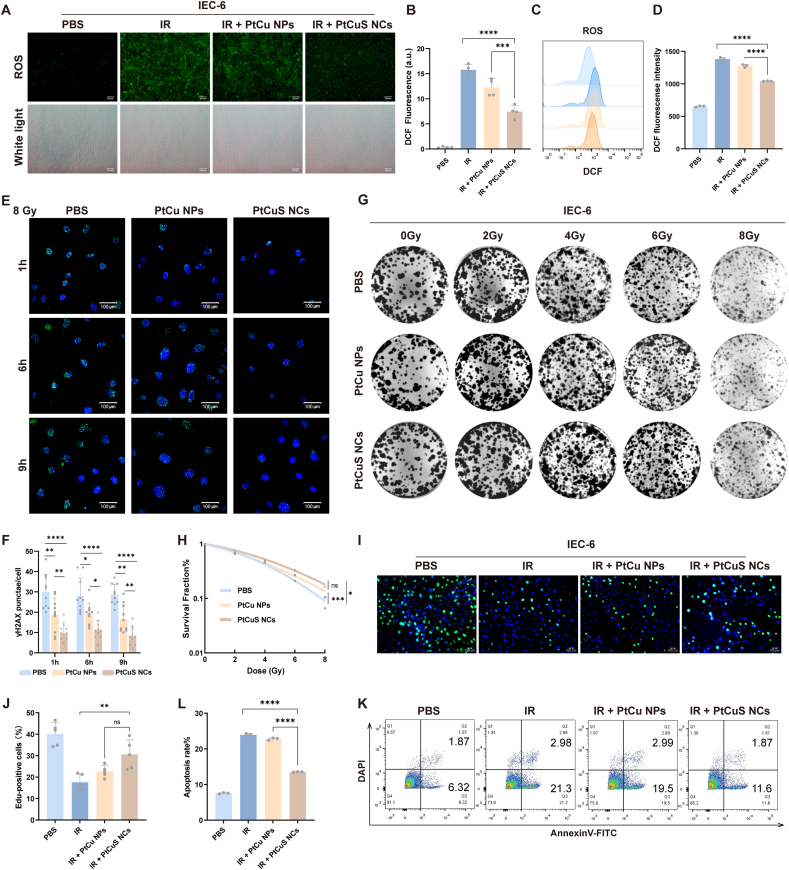
Mitigating effects of PtCuS NCs and PtCu NPs against ARIII *in vitro.* (A, B) Representative CLSM images and quantification of DCF staining in IEC-6 cells pretreated with PtCu NPs or PtCuS NCs followed by 8 Gy irradiation (*n* = 4), scale bar, 300 μm. (C, D) Representative FCM plots and quantification of DCF fluorescence intensity in IEC-6 cells (*n* = 3). (E, F) Representative images and quantification of γH2AX-Ser139 staining in IEC-6 cells pretreated with PtCu NPs or PtCuS NCs followed by 8 Gy irradiation at the indicated time points. Error bars represent SD from at least ten independent replicates. Scale bar, 100 μm. (G, H) Representative images of colony formation assays and the fitted clonogenic survival curves in IEC-6 cells (*n* = 3). (I, J) Representative EdU staining images and quantification in IEC-6 cells under the indicated treatments (*n* = 5), scale bar, 100 μm. (K, L) Representative FCM plots and quantification of apoptosis in IEC-6 cells under the indicated treatments following 8 Gy irradiation (*n* = 3). Data are presented as mean ± SD. Statistical significance was determined by one-way ANOVA followed by Tukey's multiple-comparison test for panels B, D, F, J, and L, and by nonlinear regression followed by the extra sum-of-squares F test for panel H. ∗*p* < 0.05, ∗∗*p* < 0.01, ∗∗∗*p* < 0.001, ∗∗∗∗*p* < 0.0001.

To investigate whether PtCu NPs and PtCuS NCs could scavenge radiation-induced ROS in epithelial cells, intracellular ROS production in IEC-6 and NCM460 cells was measured using 2′,7′-dichlorodihydrofluorescein diacetate (DCFH-DA), a ROS-sensitive probe. Confocal laser scanning microscopy (CLSM) and flow cytometry (FCM) indicated that the intracellular ROS levels markedly increased after 8 Gy irradiation. PtCuS NC treatment effectively reduced intracellular ROS levels, whereas PtCu NPs showed no comparable effect, indicating that PtCuS NCs can regulate intracellular redox reactions and neutralize radiation-induced ROS (Fig. 2A–D and S4A–D). To further evaluate their roles in mitigating radiation-induced damage, γH2AX, a marker of DNA damage, was detected using immunofluorescence (IF) at different time points after 8 Gy irradiation. γH2AX fluorescence intensity was lower in the PtCuS NC-treated group than in the PBS-treated group at all examined time points, suggesting that PtCuS NCs attenuated radiation-induced DNA damage (Fig. 2E, F and S4E, F).

To evaluate the radioprotective effects of PtCuS NCs on intestinal epithelial cells, colony formation assays were performed using a range of irradiation doses. After irradiation, the PtCuS NC-treated group exhibited more colonies than the PBS- and PtCu NP-treated groups (Fig. 2G and S4G). Statistical analysis of the fitted survival curves further demonstrated that treatment with PtCuS NCs conferred effective radioprotection on intestinal epithelial cells (Fig. 2H and S4H). Consistently, EdU assays showed enhanced proliferation in the PtCuS NC-treated group after 8 Gy irradiation (Fig. 2I, J and S4I, J). FCM of apoptosis showed that PtCuS NCs alleviated radiation-induced apoptosis contrasted with that in the PBS control and PtCu NPs groups (Fig. 2K, L and S4K, L). Collectively, these results indicate that PtCuS NCs protect intestinal epithelial cells from radiation-induced injury by scavenging ROS, attenuating DNA damage and apoptosis, and preserving epithelial proliferative capacity.

### 3.4 PtCuS NCs alleviate radiation-induced intestinal damage and inflammation *in vivo*

**Fig. 3:**
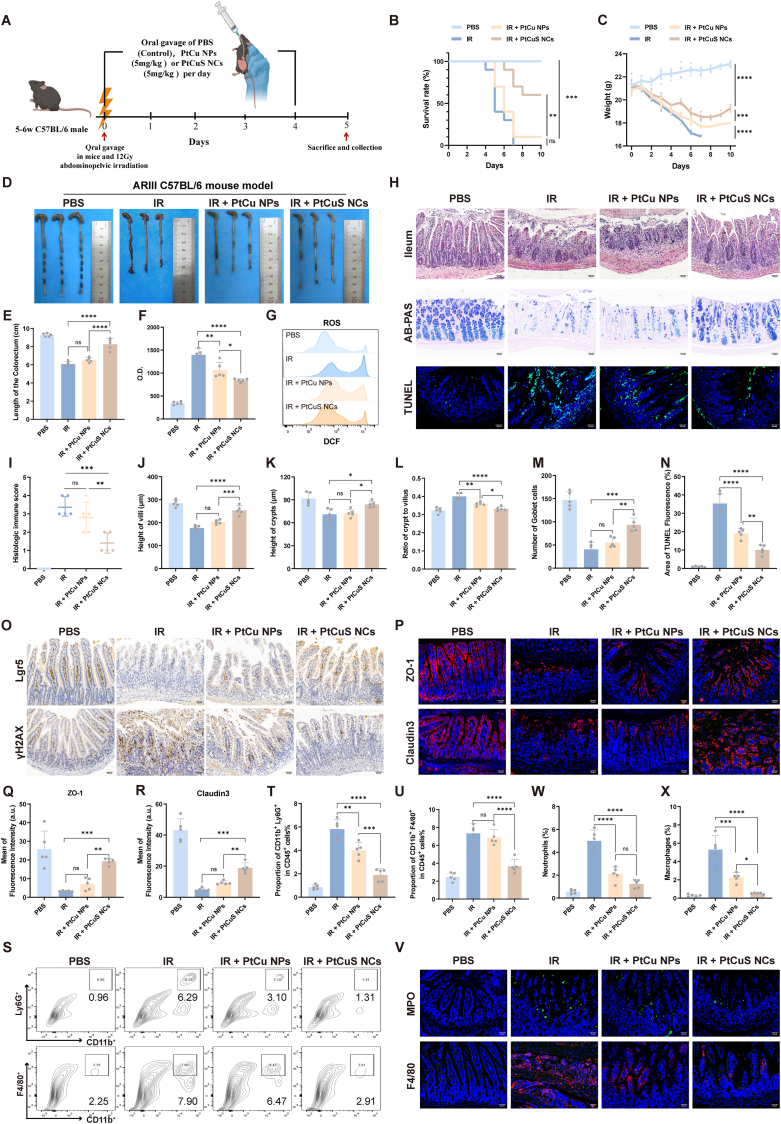
Mitigating effects of PtCuS NCs against ARIII *in vivo.* (A) Schematic illustration of the ARIII C57BL/6 mouse model with oral administration of PtCuS NCs and PtCu NPs after 12 Gy irradiation. (B, C) Survival analysis and body weight changes of C57BL/6 mice in PBS, IR, IR + PtCu NPs and IR + PtCuS NCs groups (*n* = 10)*.* (D, E) Representative necropsy images and quantification of colorectum length in the ARIII C57BL/6 mouse model (*n* = 5). (F) Quantification of serum FITC fluorescence intensity (*n* = 5). (G) FCM analysis of DCF fluorescence intensity in intestinal tissue after 12Gy irradiation (*n* = 3). (H) Representative H&E, AB-PAS, and TUNEL staining images of ileal tissues from C57BL/6 mice in the indicated groups. Scale bar, 40 μm. (I–L) Quantification of the histologic injury scores, villus height, crypt depth and the crypt-to-villus ratio in the ileum (*n* = 5). (M) Quantification of the number of goblet cells in colon (*n* = 5). (N) Quantification of TUNEL-positive area (green) (*n* = 5). (O) Representative IHC staining of Lgr5 (representing cell stemness) and γH2AX (representing cellular DNA damage) in healthy and treated mice (*n* = 5). Scale bar, 40 μm. (P–R) Representative IF staining images and quantification of the mean fluorescence intensity of ZO-1 and Claudin3 in the indicated groups (*n* = 5). Scale bar, 40 μm. (S–U) Representative FCM plots and quantification of the proportion of CD11b^+^Ly6G^+^ (neutrophils), CD11b^+^F4/80^+^ (macrophages) among CD45^+^ cells isolated from intestinal tissue (*n* = 5). (V–X) Representative IF staining images and quantification of Ly6G^+^ neutrophils and F4/80^+^ macrophages in intestinal tissue (*n* = 5). Scale bar, 40 μm. Data are presented as mean ± SD. Each dot represents one biological replicate from an individual mouse. In panels J–N, Q, R, W, and X, values for each mouse were calculated as the mean of at least three technical replicates. Survival in panel B was analyzed using Kaplan–Meier curves and compared by the log-rank (Mantel–Cox) test. Body weight changes in panel C were analyzed using a mixed-effects model with repeated measures, with treatment and time as factors. Statistical significance for panels E–G, I–N, Q, R, T, U, W, and X was determined by one-way ANOVA followed by Tukey's multiple-comparison test. Only selected statistically relevant comparisons are shown. ∗*p* < 0.05, ∗∗*p* < 0.01, ∗∗∗*p* < 0.001, ∗∗∗∗*p* < 0.0001.

Encouraged by the *in vitro* results, a C57BL/6 mouse model of ARIII was established to evaluate the radioprotective efficacy of PtCu NPs and PtCuS NCs *in vivo*. Specifically, mice were exposed to 12 Gy abdominopelvic irradiation, followed by oral administration of PtCu NPs or PtCuS NCs at 5 mg/kg for five consecutive days, and then assessed for potential intestinal injury (Fig. 3A). Comparative analysis revealed enhanced survival rates and accelerated weight recovery after PtCuS NC treatment (Fig. 3B and C). Biochemical assessments of hepatic and renal function indicators, including AST, ALT, CREA, and UREA, showed no statistically significant differences among the groups (Fig. S5A). Colorectum length was measured after treatment because it is an important indicator of intestinal injury [38]. Oral administration of PtCuS NCs after irradiation effectively preserved colorectum length, whereas PtCu NPs failed to confer comparable radioprotection (Fig. 3D and E). The FITC–dextran gavage assay was used to assess intestinal permeability, in which increased serum FITC fluorescence indicates impaired barrier integrity [32]. PtCuS NC treatment significantly reduced serum FITC–dextran leakage compared with the other groups, indicating protection against radiation-induced intestinal barrier disruption (Fig. 3F). To evaluate the ROS-scavenging activity of PtCuS NCs *in vivo*, we measured DCF fluorescence in intestinal tissues using the DCFH-DA probe. Consistent with the *in vitro* results, PtCuS NC-treated mice exhibited reduced intestinal ROS levels compared with mice in the IR alone and PtCu NP treatment groups, confirming the superior ROS-scavenging capacity *in vivo* (Fig. 3G and S5B).

To further evaluated intestinal tissue injury after irradiation, H&E staining was performed in the jejunum, ileum, and colon. Irradiation caused severe epithelial disruption, characterized by extensive villus loss and mucosal damage, whereas PtCuS NC treatment markedly alleviated these pathological changes (Fig. 3H and S5C). Quantitative analyses of the histological injury scores, villus height, and crypt depth in the ileum indicated that irradiation induced pronounced villus and crypt damage, which was effectively ameliorated by PtCuS NC treatment (Fig. 3I–K). The crypt-to-villus ratio, a recognized indicator of intestinal epithelial regeneration [31], was lower in PtCuS NC-treated mice than in PtCu NP-treated mice after irradiation, indicating improved regenerative capacity (Fig. 3L). AB-PAS staining further showed that PtCuS NC treatment protected more goblet cells after irradiation, which may help maintain intestinal barrier function by supporting mucin production (Fig. 3H, M). TUNEL staining revealed that irradiation markedly increased apoptosis in the intestinal epithelium, whereas PtCuS NCs, but not PtCu NPs, attenuated this effect (Fig. 3H, N). For further assess intestinal epithelial regeneration and irradiation-induced DNA damage, the protein levels of Lgr5 (an indicator of intestinal stemness) and γH2AX (a marker of DNA damage) were assessed. IHC analysis revealed that irradiation reduced intestinal stemness and induced DNA damage. PtCuS NCs, but not the PtCu NPs, significantly preserved stem cell populations and reduced cellular DNA damage (Fig. 3O and S5D, E). Intestinal barrier integrity was further evaluated by assessing the tight junction proteins ZO-1 and Claudin3. IF staining showed stronger ZO-1 and Claudin3 expression and more continuous junctional localization after PtCuS NC treatment than after PtCu NP treatment (Fig. 3P–R). Western blotting further confirmed increased ZO-1 and Claudin3 protein expression following PtCuS NC treatment (Fig. S5F). To directly compare the acute radioprotective effects of PtCuS NCs with those of a clinically approved agent, amifostine was included as a positive control. While amifostine partially mitigated radiation-induced body weight loss, improved survival, and moderately preserved colorectum length, villus height, crypt depth, and crypt-to-villus ratio, PtCuS NCs exhibited superior protective effects across these parameters (Fig. S6A-I). Collectively, these results indicate that PtCuS NCs outperformed the standard radioprotective agent in mitigating acute intestinal injury and maintaining epithelial integrity. Having confirmed the efficacy of PtCuS NCs in acute injury, we further assessed their impact on chronic intestinal damage. PtCuS NC-treated mice exhibited preserved colorectum length after 2 months after 10 Gy irradiation, indicating a potential long-term protective effect (Fig. S6J and K).

To clarify the anti-inflammatory effects of PtCuS NCs, we measured the levels of pro-inflammatory cytokines (IL-6, IL-1β, and TNFα) in both intestinal tissues and serum by ELISA. PtCuS NC treatment significantly reduced the levels of these cytokines compared with the IR alone and IR + PtCu NPs groups (Fig. S7A and B). Furthermore, immune cell infiltration was assessed in intestinal tissues using FCM (Fig. S7C). Irradiation markedly increased the abundance of CD11b^+^Ly6G^+^ and CD11b^+^F4/80^+^ macrophages among the CD45^+^ population, indicating enhanced recruitment of neutrophils and macrophages to injured intestinal tissue. Notably, PtCuS NC treatment reduced the infiltration of both neutrophils and macrophages (Fig. 3S–U). Consistent with the FCM results, IF analysis further indicated that treatment with PtCuS NCs reduced neutrophil and macrophage infiltration (Fig. 3V–X). Collectively, these results demonstrate that PtCuS NCs effectively alleviated radiation-induced intestinal inflammation *in vivo*.

### 3.5 Investigation of the mechanism of PtCuS NCs in relieving ARIII

**Fig. 4:**
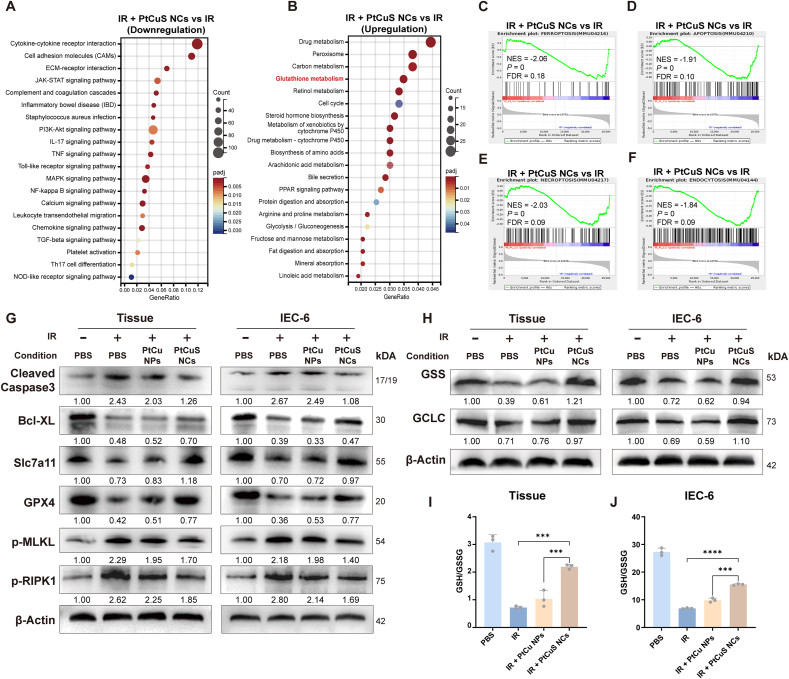
Mechanisms by which PtCuS NCs alleviate ARIII. (A, B) KEGG enrichment analysis of the genes downregulated (A) and upregulated (B) in intestinal tissues from the IR + PtCuS NCs group compared with the IR group. (C–F) GSEA enrichment analysis of the pathways including ferroptosis, apoptosis, necroptosis, and endocytosis. (G) Western blot analysis of death-related proteins including Bcl-XL, Cleaved Caspase3, GPX4, Slc7a11, p-MLKL, and p-RIPK1 in indicated groups after 12Gy irradiation *in vivo* and 8 Gy irradiation *in vitro* (IEC-6). (H) Western blot was used to detect the protein expression levels of GSS and GCLC *in vitro* and *in vivo*. (I, J) Measurement of the ratio of reduced glutathione (GSH) to oxidized glutathione (GSSG) in intestinal tissues and IEC-6, respectively (*n* = 3). Data are presented as mean ± SD. Each dot represents one biological replicate from an individual mouse in panel I. Statistical significance for panels I and J was determined by one-way ANOVA followed by Tukey's multiple-comparison test. ∗*p* < 0.05, ∗∗*p* < 0.01, ∗∗∗*p* < 0.001, ∗∗∗∗*p* < 0.0001.

To explore the underlying mechanism responsible for the benefits of PtCuS NCs in ARIII, RNA-seq was performed on the intestinal tissues from C57BL/6 mice in the IR and IR + PtCuS NCs groups. Volcano plot analysis revealed marked differential gene expression between the two groups, with 1714 significantly upregulated genes and 2693 significantly downregulated genes in the IR + PtCuS NCs group compared with the IR group (Fig. S8A). Heatmap cluster analysis further showed distinct gene expression profiles between the two groups (Fig. S8B). Kyoto Encyclopedia of Genes and Genomes (KEGG) pathway analysis of the differentially expressed genes showed that pro-inflammatory signaling pathways, including the PI3K–AKT, IL-17, TNF, Toll-like receptor, and NF-κB signaling pathways, were enriched in the IR group relative to the IR + PtCuS NCs group (Fig. 4A). Consistently, PtCuS NC treatment reduced the expression of key pro-inflammatory genes, including *IL-1β, Cxcl5, Cxcl1, IL-6,* and *TNF* (Fig. S8C). In parallel, several metabolic pathways, including glutathione (GSH) metabolism, retinol metabolism, amino acid biosynthesis, glycolysis, and fat digestion and absorption, were enriched in the IR + PtCuS NCs group, suggesting an association with antioxidant defense and metabolic recovery (Fig. 4B). Gene Ontology (GO) enrichment analysis further indicated that the differentially expressed genes were associated with fatty acid metabolism, peroxisome function, and oxidoreductase activity, consistent with improved antioxidant capacity and intestinal repair after PtCuS NC treatment (Fig. S8D). Given that cell death is a key terminal event in radiation-induced intestinal injury, we further performed Gene Set Enrichment Analysis (GSEA) to evaluate relevant cell death pathways. PtCuS NC treatment was negatively associated with key cell death pathways, including ferroptosis, apoptosis, and necroptosis (Fig. 4C–E). In addition, endocytosis, a functional pathway related to immune cell activity, also showed negative enrichment in the PtCuS NC-treated group (Fig. 4F).

Western blotting was performed in intestinal epithelial cells and tissues to validate the effects of PtCuS NCs on cell death pathways. Irradiation markedly reduced the levels of the ferroptosis-related proteins Slc7a11 and GPX4, the anti-apoptotic protein Bcl-XL, while increasing the levels of pro-apoptotic cleaved Caspase3 and key necroptosis-related proteins, including p-MLKL and p-RIPK1. PtCuS NC treatment restored the expression of Slc7a11, GPX4, and Bcl-XL, and reduced cleaved Caspase3, p-MLKL, and p-RIPK1 expression, suggesting that PtCuS NCs attenuate radiation-induced ferroptosis, apoptosis, and necroptosis (Fig. 4G and S8E).

### 3.6 PtCuS NCs promote the GSH metabolism pathway to alleviate radiation-induced cell death and inflammation

GSH metabolism pathway is essential for maintaining cellular redox homeostasis and plays an important role in limiting cellular ferroptosis, apoptosis, and necroptosis [[39], [40], [41]]. Consistent with the transcriptomic and KEGG enrichment analyses, qPCR validation in both *in vivo* and *in vitro* models showed increased expression of key GSH biosynthesis genes, including *GSS* and *GCLC*, after PtCuS NC treatment (Fig. S9A-C). Further validation at both the cellular and tissue levels showed that PtCuS NC treatment after irradiation increased the protein expression of GSS and GCLC and elevated the GSH/GSSG ratio (Fig. 4H–J and S9D, E).

**Fig. 5:**
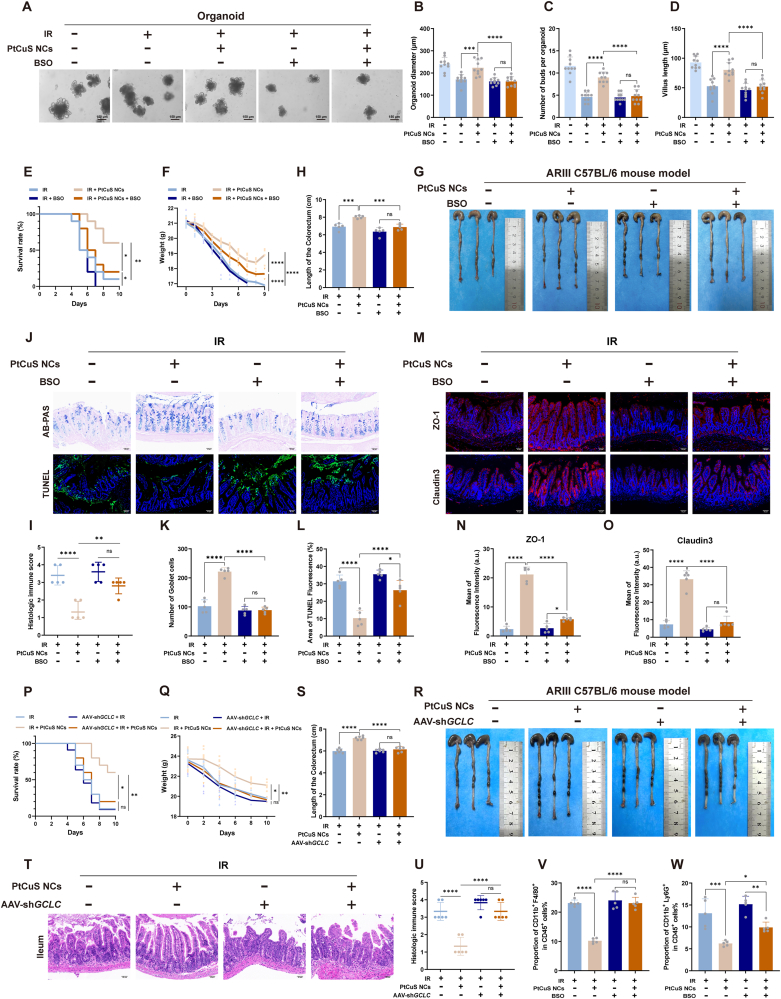
Effects and mechanisms of PtCuS NCs in alleviating radiation-induced intestinal inflammation and injury. (A).Representative bright-field images of small intestinal organoids. Scale bar, 150 μm. (B–D) Quantification of organoid diameter, bud number per organoid and villus length (*n* = 10). (E, F) Survival analysis and body weight changes of C57BL/6 mice treated with or without PtCuS NCs and BSO (*n* = 10)*.* (G, H) Representative necropsy and quantification of colorectum length in the indicated groups after 12 Gy irradiation (*n* = 5). (I) Quantification of the histologic immunity scores (*n* = 5). (J–L) Representative images and quantification of AB-PAS and TUNEL staining in C57BL/6 mice from the indicated groups. Scale bar, 40 μm. (M − O) Representative IF image and quantification of mean fluorescence intensity of ZO-1 and Claudin3 in indicated group (*n* = 5). Scale bar, 40 μm. (P, Q) Survival analysis and body weight changes of C57BL/6 mice with or without PtCuS NCs and AAV-sh*GCLC* (*n* = 10)*.* (R, S) Representative necropsy images and quantification of colorectum length in the indicated groups after 12 Gy irradiation (*n* = 6). (T, U) Representative H&E staining images of ileal tissues and quantification of histological injury scores (*n* = 6). (V, W) FCM quantification of the proportion of CD11b^+^Ly6G^+^ (neutrophils) and CD11b^+^F4/80^+^ (macrophages) among CD45^+^ cells isolated from intestinal tissue in mice treated with or without PtCuS NCs and BSO (*n* = 5). Data are presented as mean ± SD. For the *in vivo* experiments, each dot represents one biological replicate from an individual mouse. In panels K, L, N, and O, values for each mouse were calculated as the mean of at least three technical replicates. Survival in panels E and P was analyzed using Kaplan–Meier curves and compared by the log-rank (Mantel–Cox) test. Body weight changes in panels F and Q were analyzed using a mixed-effects model with repeated measures. Statistical significance for panels B–D, H, I, K, L, N, O, S, U, V, and W was determined by two-way ANOVA followed by Tukey's multiple-comparison test. ∗*p* < 0.05, ∗∗*p* < 0.01, ∗∗∗*p* < 0.001, ∗∗∗∗*p* < 0.0001.

To elucidate whether GSH-related metabolism contributes to PtCuS NC-mediated protection against radiation-induced injury, we introduced BSO, a specific inhibitor of GSH synthesis. *In vitro*, IEC-6 and NCM460 cells were pretreated with 100 μM BSO for 24 h before irradiation. Colony formation assays showed that BSO attenuated the radioprotective effect of PtCuS NCs, supporting the role of GSH metabolism in mediating the protective activity (Fig. S10A-D). EdU assays revealed that the proliferative capacity preserved by PtCuS NCs was reduced in the presence of BSO (Fig. S10E-H). FCM analysis further demonstrated that BSO impaired the ability of PtCuS NCs to suppress apoptosis after irradiation (Fig. S10I-K). To examine the effect of BSO on PtCuS NC-mediated regulation of cell death-related pathways, western blotting was performed. BSO treatment reduced the levels of the ferroptosis-related proteins Slc7a11 and GPX4 and the anti-apoptotic protein Bcl-XL, while increasing cleaved Caspase3, p-MLKL, and p-RIPK1 in PtCuS NC-treated cells (Fig. S10L). These results suggest that GSH-related metabolism contributes to the cytoprotective effects of PtCuS NCs *in vitro*. Furthermore, mouse small intestinal organoids showed that PtCuS NCs increased organoid diameter, villus length, and bud number, whereas these protective effects were largely attenuated by BSO treatment, further supporting the involvement of GSH-related metabolism in PtCuS NC-mediated intestinal epithelial protection (Fig. 5A–D).

*In vivo*, we established a C57BL/6 mouse model of ARIII and divided the mice into four treatment groups: (i) IR, (ii) IR + PtCuS NCs (5 mg/kg), (iii) IR + BSO (5 mmol/kg in distilled water, 10 mL/kg), and (iv) IR + PtCuS NCs + BSO. Compared with the IR + PtCuS NCs group, mice in the IR + PtCuS NCs + BSO group exhibited reduced survival, greater weight loss, and shorter colorectum length (Fig. 5E–H). H&E staining revealed that the PtCuS NC treatment preserved intestinal morphology after irradiation, whereas BSO co-treatment weakened this protection, as reflected by increased histological injury scores, reduced villus height and crypt depth, and an increased crypt-to-villus ratio (Fig. 5I and S11A-D). AB-PAS staining further showed that BSO attenuated the protective effect of PtCuS NCs on goblet cells (Fig. 5J and K). To further evaluate whether GSH-related metabolism contributes to PtCuS NC-mediated protection, TUNEL staining and western blotting were performed. BSO reduced the ability of PtCuS NCs to alleviate radiation-induced apoptosis and weakened their regulatory effects on ferroptosis, apoptosis, and necroptosis markers *in vivo* (Fig. 5J, L and S10L). Additionally, IHC analysis of Lgr5 and γH2AX indicated that BSO compromised the effects of PtCuS NCs on intestinal stem cell preservation and DNA damage reduction (Fig. S11E-G). Moreover, IF and Western blotting of the tight junction proteins ZO-1 and Claudin3 revealed decreased expression in the IR + PtCuS NCs + BSO group compared with the IR + PtCuS NCs group, indicating that BSO impaired the barrier-preserving effect of PtCuS NCs (Fig. 5M-O and Fig. S11H).

Given that GCLC is the rate-limiting enzyme in GSH synthesis, we used AAV-mediated *GCLC* knockdown to evaluate the contribution of GSH metabolism to PtCuS NC-mediated intestinal protection. Knockdown efficiency was verified in mouse intestinal tissue by qPCR and western blotting (Fig. S11I and J). In these mice, core measurements, including survival, body weight, colorectum length, and histological injury, showed that the protective effects of PtCuS NCs were markedly attenuated, consistent with the BSO results (Fig. 5P–U and S11K–N). Together, these findings support an important role of GSH-related metabolism in PtCuS NC-mediated protection against radiation-induced intestinal injury.

Radiation-induced cell death is closely associated with the release of inflammatory factors, including IL-6, IL-1β and TNFα. Analysis of mouse intestinal tissues and serum using ELISA showed that PtCuS NC treatment reduced the levels of these inflammatory cytokines, whereas this effect was largely weakened by BSO co-administration (Fig. S12A and B). Consistently, qPCR analysis showed that BSO partially attenuated the anti-inflammatory effects of PtCuS NCs in intestinal tissue (Fig. S12C). FCM analysis revealed that increased infiltration of neutrophil (CD11b^+^Ly6G^+^) and macrophage (CD11b^+^F4/80^+^) in the IR + PtCuS NCs + BSO group compared with the IR + PtCuS NCs group (Fig. 5V, W and Fig. S12D and E). IF analysis further confirmed increased infiltration of these immune cells (Fig. S12F-H). These results suggest that GSH-related metabolism contributes to the anti-inflammatory effects of PtCuS NCs, potentially through attenuation of radiation-induced cell death and subsequent inflammatory cell recruitment.

### 3.7 PtCuS NCs modulate macrophage polarization to exert anti-inflammatory effects

**Fig. 6:**
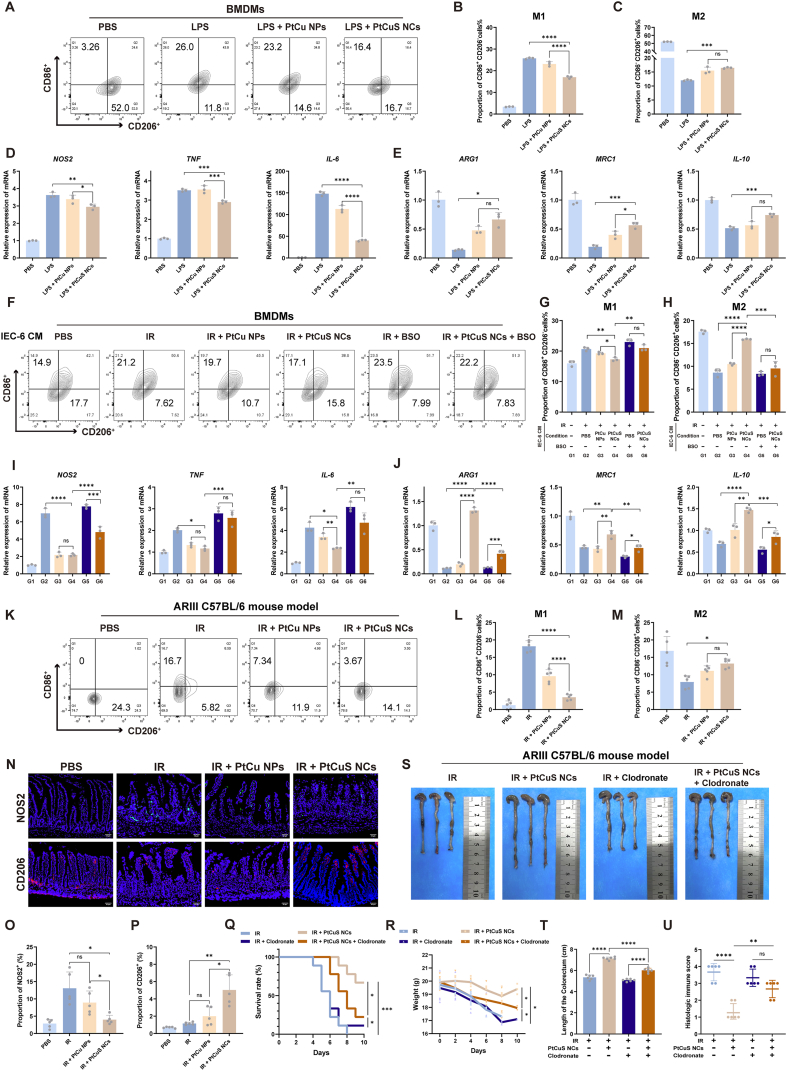
PtCuS NCs modulate macrophage polarization to exert anti-inflammatory effects. (A–C) Representative FCM plots and quantification of M1-like CD86^+^CD206^-^ and M2-like CD86^−^CD206^+^ BMDMs in the indicated groups (*n* = 3). (D, E) qPCR analysis of M1-like markers(*NOS2*, *TNF*, and *IL-6*) and M2-like markers (*ARG1*, *MRC1*, and *IL-10*) in BMDMs from the indicated groups (*n* = 3). (F–H) Representative FCM plots and quantification of M1-like CD86^+^CD206^-^ and M2-like CD86^−^CD206^+^ BMDMs primed with low-dose LPS (10 ng/mL) and then cultured in conditioned medium (CM) from IEC-6 cells exposed to different treatments, including PBS, IR, IR + PtCu NPs, IR + PtCuS NCs, IR + BSO, and IR + PtCuS NCs + BSO (*n* = 3). (I, J) qPCR of M1-like markers (*NOS2*, *TNF* and *IL-6*) and M2-like markers (*ARG1*, *MRC1* and *IL-10*) from BMDMs in indicated groups (*n* = 3). (K–M) Representative FCM plots and quantification of the proportions of M1-like CD86^+^CD206^-^ and M2-like CD86^−^CD206^+^ macrophages isolated from intestinal tissues in the indicated groups (*n* = 5). (N–P) Representative IF staining images and quantification of NOS2 and CD206 in intestinal tissue (*n* = 5). Scale bar, 40 μm. (Q, R) Survival analysis and body weight changes C57BL/6 mice with or without PtCuS NCs and Clodronate (*n* = 9). (S, T) Representative necropsy images and quantification of colorectum length in the indicated groups after 12 Gy irradiation (*n* = 6). (U) Quantification of the histologic immunity scores (*n* = 6). Data are presented as mean ± SD. For the *in vivo* experiments, each dot represents one biological replicate from an individual mouse. Statistical significance for panels B–E, L, M, O, and P was determined by one-way ANOVA followed by Tukey's multiple-comparison test. For panels G–J, one-way ANOVA followed by Tukey's multiple-comparison test was used for comparisons among PBS, IR, IR + PtCu NPs, and IR + PtCuS NCs, whereas two-way ANOVA followed by Tukey's multiple-comparison test was used for BSO-related comparisons among IR, IR + PtCuS NCs, IR + BSO, and IR + PtCuS NCs + BSO. Survival in panel Q was analyzed using Kaplan–Meier curves and compared by the log-rank (Mantel–Cox) test. Body weight changes in panel R were analyzed using a mixed-effects model with repeated measures. Statistical significance for panels T and U was determined by two-way ANOVA followed by Tukey's multiple-comparison test. ∗*p* < 0.05, ∗∗*p* < 0.01, ∗∗∗*p* < 0.001, ∗∗∗∗*p* < 0.0001.

Given the central role of macrophages in mediating inflammatory responses after radiation [42,43], we next investigated whether PtCuS NCs could modulate macrophage polarization to exert anti-inflammatory effects. We first assessed the direct regulatory capacity of PtCuS NCs on macrophages. Bone marrow-derived macrophages (BMDMs) were isolated from 6-week-old male C57BL/6 mice and maintained in an unstimulated M0-like state. FCM results showed that co-incubation with PtCuS NCs significantly reduced the proportion of LPS (lipopolysaccharide)-induced M1-like pro-inflammatory macrophages (CD86^+^CD206^-^) and increased the population of M2-like reparative macrophages (CD86^−^CD206^+^) (Fig. S13A-D). Consistently, qPCR analysis showed reduced expression of M1-related markers (*iNOS*, *TNF*, and *IL-6*) and increased expression of M2-related markers (*ARG1*, *MRC1*, and *IL-10*) (Fig. S13E and F). Notably, FCM and qPCR analyses further revealed that PtCuS NCs also shifted pre-induced M1-like macrophages toward a less inflammatory and more reparative phenotype, supporting their direct role in macrophage reprogramming (Fig. 6A–E).

Furthermore, since damage-associated molecular patterns (DAMPs) and inflammatory factors released by injured intestinal tissues could promote M1-like polarization [44], we therefore examined whether PtCuS NCs could indirectly regulate macrophage polarization by modifying the irradiated intestinal epithelial microenvironment. For this purpose, M0-like BMDMs were primed with low-dose LPS (10 ng/mL) and then cultured in the conditioned medium (CM) collected from IEC-6 cells exposed to different treatments. Compared with CM from irradiated IEC-6 cells, CM from PtCuS NC-treated irradiated IEC-6 cells reduced microenvironment-driven M1-like polarization and promoted a shift toward an M2-like phenotype (Fig. 6F–H). This effect was partially reversed by BSO, with both M1-and M2-associated marker profiles shifting closer to those observed in the IR group (Fig. 6F–H). qPCR analysis of M1-and M2-associated markers in the corresponding groups further supported these findings (Fig. 6I and J). These results suggest that PtCuS NCs regulate macrophage polarization through direct effects on macrophages and indirect effects mediated by the irradiated epithelial microenvironment, in which epithelial GSH-related metabolism may contribute.

Encouraged by the *in vitro* findings, we further evaluated the effects of PtCuS NCs on macrophage phenotypes in the ARIII mouse model. FCM analysis of intestinal tissues revealed that abdominopelvic irradiation significantly elevated the M1-like macrophage population, whereas PtCuS NC administration reduced the M1-like macrophage population and elevated the M2-like macrophage population (Fig. 6K–M). IF staining of NOS2 and CD206 also indicated a decreased proportion of M1-like macrophages and an increased proportion of M2-like macrophages after PtCuS NC administration (Fig. 6N–P). To determine whether macrophage modulation contributes to the anti-inflammatory and radioprotective effects of PtCuS NCs, macrophages were systemically depleted by intraperitoneal injection of clodronate liposomes before and after irradiation. Macrophage depletion undermined the protective effects of PtCuS NCs against ARIII, as evidenced by reduced survival rates, greater body weight loss, and shorter colorectum length (Fig. 6Q–T). Histological evaluations via H&E staining showed that clodronate treatment minimized the differences in histological injury scores, ileal villus height, crypt depth, crypt-to-villus ratio, and colonic goblet cell counts between the IR and IR + PtCuS NCs groups (Fig. 6U and Fig. S13G-J). Notably, PtCuS NC treatment still elevated the epithelial GSH/GSSG ratio in intestinal tissues after macrophage depletion (Fig. S13K), indicating that PtCuS NCs could enhance intestinal antioxidant status independently of macrophage-derived signals. These results support an important role of macrophage polarization in PtCuS NC-mediated alleviation of intestinal inflammation.

### 3.8 PtCuS NCs attenuate gut microbiota dysbiosis and modulate metabolites after irradiation

**Fig. 7:**
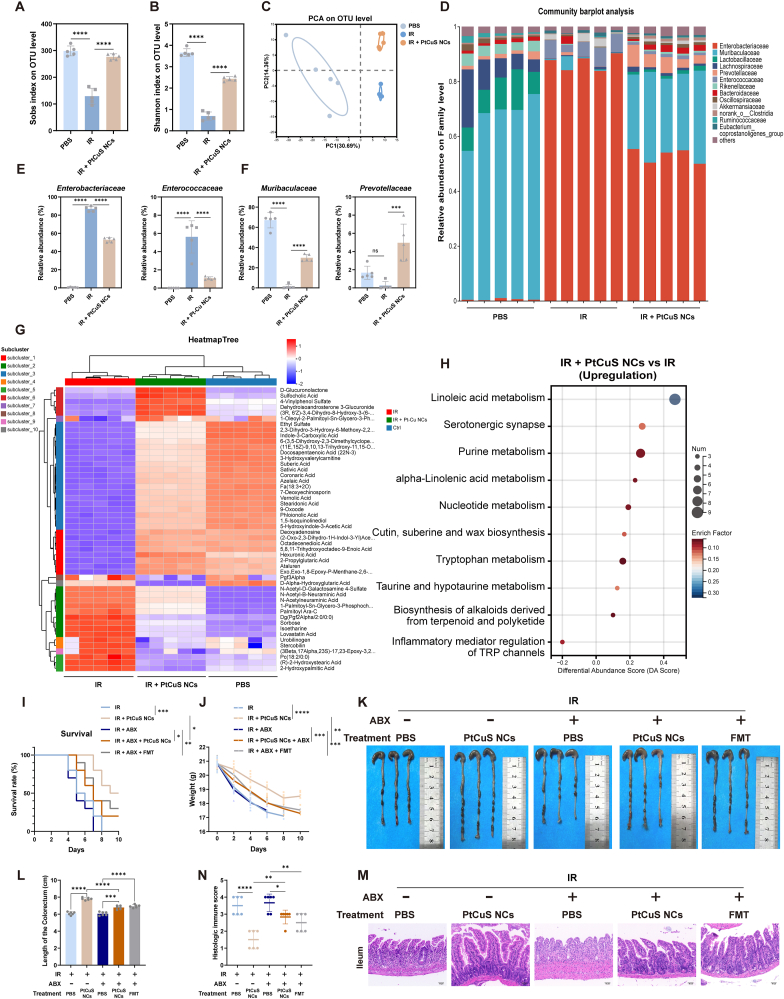
PtCuS NCs attenuate gut microbiota dysbiosis and modulate metabolites. (A, B) Sobs and Shannon indices of fecal microbiota in the indicated groups (*n* = 5). (C) PCA analysis of fecal microbiota in the indicated groups (*n* = 5). (D) Taxonomic composition analysis at the family level. (E) Relative abundance of *Enterobacteriaceae* and *Enterococcaceae* in fecal microbiota from the indicated groups (*n* = 5). (F) Relative abundance of *Muribaculaceae* and *Prevotellaceae* in fecal microbiota from the indicated groups (*n* = 5). (G) Heatmap analysis of metabolites among groups. (H) KEGG enrichment analysis of the differential metabolites among groups. (I, J) Survival analysis and body weight changes of C57BL/6 mice with or without ABX and with PtCuS NCs or FMT derived from IR + PtCuS NC-treated donors (*n* = 10). (K, L) Representative necropsy images and quantification of colorectum length in the indicated groups after 12 Gy irradiation (*n* = 6). (M, N) Representative H&E staining images of ileal tissues and quantification of histological injury scores (*n* = 6). Data are presented as mean ± SD. For the *in vivo* experiments, each dot represents one biological replicate from an individual mouse. Statistical significance for panels A, B, E, and F was determined by one-way ANOVA followed by Tukey's multiple-comparison test. Survival in panel I was analyzed using Kaplan–Meier curves and compared by the log-rank (Mantel–Cox) test. Body weight changes in panel J were analyzed using a mixed-effects model with repeated measures. For panels L and N, two-way ANOVA followed by Tukey's multiple-comparison test was used for comparisons among the IR, IR + PtCuS NCs, IR + ABX, and IR + ABX + PtCuS NCs groups, whereas one-way ANOVA followed by Tukey's multiple-comparison test was used for comparisons among the IR, IR + ABX, and IR + ABX + FMT groups. ∗*p* < 0.05, ∗∗*p* < 0.01, ∗∗∗*p* < 0.001, ∗∗∗∗*p* < 0.0001.

The gut microbiota plays a critical role in intestinal disorders, including radiation-induced injury [45]. Moreover, ROS can function as electron acceptors, facilitating anaerobic respiration and the expansion of pathogenic anaerobic bacteria [46]. The ROS arising from irradiation can disrupt the microbial equilibrium, ultimately contributing to gut dysbiosis [47]. To investigate whether PtCuS NCs modulate gut microbiota, comprehensive *16S* rRNA sequencing analysis of fecal samples was performed. The analysis revealed significant alterations in gut microbiota composition and structure among the groups (Fig. S14A). Irradiation markedly reduced the gut microbial alpha diversity, as evidenced by decreased Sobs (Observed Species) and ACE (Abundance-based Coverage Estimator) indices, whereas PtCuS NC treatment restored community richness toward near-normal levels (Fig. 7A and S14B). Additionally, PtCuS NC treatment increased microbial community diversity, as indicated by an enhanced Shannon index and reduced Simpson index (Fig. 7B and S14C). Principal component analysis (PCA) and principal coordinate analysis (PCoA) showed clear separation of the microbial communities among the PBS, IR, and IR + PtCuS NCs groups, indicating distinct beta diversity patterns (Fig. 7C and S14D). Both the Microbial Dysbiosis Index (MDI) and Gut Microbiome Health Index (GMHI) suggested partial restoration of a healthier microbiota after PtCuS NC treatment (Fig. S14E and F). Furthermore, taxonomic composition analyses at the phylum and family levels revealed marked differences in microbial communities among these groups (Fig. 7D and S14G). At the phylum level, irradiation increased the relative abundance of *Proteobacteria* and decreased that of *Bacteroidota*, whereas PtCuS NC treatment largely reversed these changes (Fig. S14H). At the family level, beneficial *Muribaculaceae* and *Prevotellaceae*, known to produce short-chain fatty acids and inhibit pro-inflammatory cytokines [48,49], were elevated, whereas potentially pathogenic or inflammation-associated taxa, including *Enterobacteriaceae* and *Enterococcaceae*, which may promote intestinal inflammation [50,51], were reduced after PtCuS NC treatment (Fig. 7E and F). Consistently, the circular phylogenetic tree showed that taxa enriched after PtCuS NC treatment included potentially beneficial families such as *Muribaculaceae*, *Oscillospiraceae*, and *Lachnospiraceae* (Fig. S14I).

Given the gut microbiota's role in host physiology through metabolite production [52], untargeted fecal metabolomics based on LC–MS was performed. PtCuS NC treatment significantly altered 475 metabolites compared with IR alone (350 upregulated, 125 downregulated; Fig. S14J). Specifically, PtCuS NCs increased the level of short-chain fatty acid derivatives, tryptophan catabolites, and indole derivatives, which are associated with intestinal repair and inflammation resolution, while reducing pro-inflammatory metabolites such as prostaglandin F2α, LysoPC, and N-acetylneuraminic acid [[53], [54], [55], [56], [57]] (Fig. 7G). KEGG enrichment analysis consistently revealed that PtCuS NCs was associated with enhanced nutrition and anti-inflammation pathways and reduced the pro-inflammatory pathways (Fig. 7H).

To examine the role of gut microbiota in PtCuS NC-mediated protection, we depleted intestinal bacteria using a broad-spectrum antibiotic cocktail (ABX) and performed fecal microbiota transplantation (FMT) using donors treated with IR + PtCuS NCs. ABX treatment partially compromised PtCuS NC-mediated radioprotection, as evidenced by impaired in survival, body weight, colorectum length, and other radioprotection-associated indices. FMT from IR + PtCuS NCs donors partially restored these protective outcomes in ABX-treated mice; however, the degree of protection remained lower than that achieved by PtCuS NC treatment in the absence of ABX (Fig. 7I–N and S14K–N). These results suggest that the gut microbiota functionally contributes to, but does not fully account for, the radioprotective effects of PtCuS NCs, highlighting the importance of microbial composition and metabolite modulation in radioprotective mechanism.

### 3.9 PtCuS NCs do not aggravate cancer progression and diminish radiotherapy efficacy

**Fig. 8:**
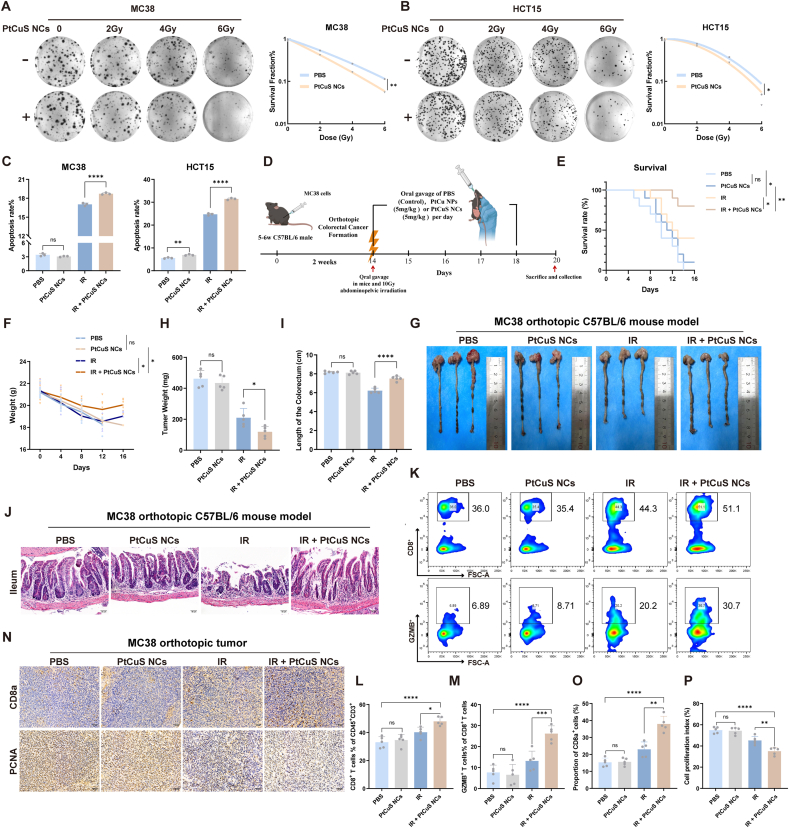
PtCuS NCs do not diminish radiotherapy efficacy. (A, B) Representative images of colony formation assays and fitted clonogenic survival curves in MC38 and HCT15 cells treated with or without PtCuS NCs following irradiation at the indicated doses (*n* = 3). (C) Quantification of apoptosis in MC38 and HCT15 cells treated with or without PtCuS NCs and following 8Gy irradiation (*n* = 3). (D) Schematic illustration of the MC38 orthotopic tumor model in C57BL/6 mice with oral administration of PtCuS NCs after 12Gy irradiation. (E, F) Survival analysis and body weight changes of MC38 orthotopic tumor-bearing C57BL/6 mice treated with or without IR and PtCuS NCs (*n* = 10). (G–I) Representative necropsy images and quantification of tumor weight and colorectum length in MC38 orthotopic tumor-bearing mice (*n* = 5). (K–M) Representative FCM plots and quantification of the proportion of CD45^+^CD3^+^CD8^+^ T cells and CD45^+^CD3^+^CD8^+^GZMB^+^ effector T cells in tumor tissues (*n* = 5). (N–P) Representative IHC staining and quantification of CD8a and PCNA (representing cell proliferation) (*n* = 5). Scale bar, 40 μm. Data are presented as mean ± SD. For *in vivo* experiments, each dot represents one biological replicate from an individual mouse. In panels O and P, values for each mouse were calculated as the mean of at least three technical replicates. Statistical significance for panels A and B was determined by nonlinear regression followed by the extra sum-of-squares F test. Statistical significance for panels C, H, I, L, M, O, and P was determined by two-way ANOVA followed by Tukey's multiple-comparison test. Survival in panel E was analyzed using Kaplan–Meier curves and compared by the log-rank (Mantel–Cox) test. Body weight changes in panel F were analyzed using a mixed-effects model with repeated measures. ∗*p* < 0.05, ∗∗*p* < 0.01, ∗∗∗*p* < 0.001, ∗∗∗∗*p* < 0.0001.

The efficacy of PtCuS NCs in mitigating ARIII was demonstrated in the aforementioned experiments. We therefore conducted a series of oncology-related experiments to evaluate their translational potential in colorectal cancer radiotherapy. *In vitro*, colony formation assays were performed on human colorectal cancer cells, DLD-1 and HCT15, and murine colorectal cancer cells, MC38 and CT26. PtCuS NC treatment alone did not increase clonogenic growth in these colorectal cancer cell lines. After irradiation, the PtCuS NC-treated groups formed fewer colonies than the PBS-treated groups (Fig. 8A, B and S15A, B). Statistical analysis of the cell survival rates and fitted curves demonstrated that PtCuS NC treatment did not compromise radiation-induced suppression of clonogenic survival and tended to enhance the radiosensitivity of colorectal cancer cells. FCM analysis further showed that PtCuS NCs modestly increased apoptosis in colorectal cancer cells compared with irradiation alone (Fig. 8C and S15C, D).

*In vivo* MC38 orthotopic tumor models were established in male C57BL/6 mice. Two weeks after tumor implantation, mice were randomly assigned to four experimental groups: PBS control, PtCuS NCs alone, IR alone (10 Gy abdominopelvic irradiation), and IR + PtCuS NCs (Fig. 8D). PtCuS NCs were administered by oral gavage for five consecutive days. To assess the long-term outcomes and overall safety of PtCuS NC treatment, mouse survival and body weight were monitored over time. PtCuS NC treatment alone did not increase mortality or cause significant body weight loss compared with the PBS control group (Fig. 8E and F). Moreover, mice in the IR + PtCuS NCs group exhibited a slight increase in survival and body weight compared with the IR-alone group, indicating that PtCuS NCs did not compromise radiotherapy efficacy while providing a favorable safety profile (Fig. 8E and F). Necropsy images confirmed that PtCuS NCs alone did not promote tumor growth compared with the PBS control group (Fig. 8G and H). The combination of PtCuS NCs with IR reduced tumor weight while preserving colorectum length (Fig. 8G–I). H&E staining of the jejunum, ileum, and colon, and histological injury scores revealed that the PtCuS NC treatment preserved intestinal morphology after irradiation (Fig. 8J and Fig. S15E, F). Quantitative measurements of villus height, crypt depth, and crypt-to-villus ratio further confirmed the protective effect of PtCuS NCs against ARIII (Fig. S15G–I).

To investigate potential immune effects, FCM of CD8^+^ T cells in the tumor tissues was performed (Fig. S15J). The IR + PtCuS NCs group exhibited a slight increase in CD45^+^CD3^+^CD8^+^ T cells and GZMB^+^ effector CD8^+^ T cells (Fig. 8K–M), and IHC analysis further confirmed modestly higher CD8^+^ T cell infiltration (Fig. 8N and O). IHC analysis also showed reduced PCNA expression, suggesting a slight decrease in tumor cell proliferative activity (Fig. 8N, P). Collectively, these results demonstrate that PtCuS NCs do not promote tumor progression and may modestly modulate the tumor immune microenvironment, highlighting their potential to reduce ARIII without compromising antitumor efficacy.

## 4 Discussion

ARIII remains a major clinical challenge in patients with locally advanced rectal cancer [58]. Despite advances in radiation techniques, oxidative stress, epithelial injury, and intestinal dysbiosis continue to undermine the patients’ quality of life and therapeutic outcomes [59]. Currently, there are no FDA-approved pharmacological interventions specifically for ARIII. Therefore, there is a critical need to develop therapeutic strategies to mitigate intestinal injury without compromising antitumor efficacy.

Recent reviews have highlighted several advantages of nanozymes over natural enzymes, including higher stability and multiple enzyme-like activities [60]. These properties enable broad applications in ROS regulation, inflammation control, and tissue repair, further supporting their potential in biomedical interventions. Motivated by the lack of effective therapies for ARIII, we developed PtCu-based nanozymes for mitigation ARIII. Amorphous PtCuS NCs are smaller and more monodisperse than crystalline PtCu NPs. HAADF-STEM and EDX measurements confirmed the uniform distribution of Pt and Cu atoms, providing a structural basis for bimetallic cooperation. Sulfur incorporation altered the local electronic environment of Pt, as reflected by the shift in Pt 4f binding energy and the presence of Pt(0) and Pt(+2) states in XPS measurements, potentially enhancing Pt-Cu synergistic catalysis. These combined structural and electronic features contributed to higher CAT- and SOD-like activities, stronger substrate affinity, and faster catalytic turnover compared with PtCu NPs. In addition, PtCuS NCs demonstrated favorable biocompatibility under the tested conditions, with negligible cytotoxicity and no observed organ damage. Importantly, the small size, negative surface charge, and structural stability of PtCuS NCs may contribute to their enhanced intestinal retention after oral administration, which is beneficial for sustained local ROS scavenging at injured intestinal sites. Collectively, the combination of bimetallic synergy, amorphous nanocluster architecture, and sulfur-mediated electronic modulation provides a rational design strategy for mitigating ARIII.

Excessive ROS generation following irradiation triggers cellular injury, intestinal integrity disruption, and inflammatory cascades [61]. PtCuS NCs effectively scavenged ROS and protected intestinal tissues from radiation-induced damage and inflammation. Consistently, intracellular ROS levels, DNA damage, and subsequent cell death, including apoptosis, ferroptosis, and necroptosis, were significantly reduced, whereas epithelial cell proliferation, intestinal barrier integrity, and overall survival were improved. In the *in vivo* model, PtCuS NCs showed more pronounced protection than the commonly used radioprotective agent amifostine under the respective treatment regimens. This improved protection may reflect the intrinsic ROS-scavenging activity and intestinal retention of PtCuS NCs, together with route-dependent biodistribution and local intestinal exposure. Mechanistically, PtCuS NCs ameliorated disruptions in GSH-related metabolism, a critical system for redox homeostasis and cell survival. BSO-mediated glutathione depletion attenuated the protective effects of PtCuS NCs *in vivo*, including the suppression of apoptosis, ferroptosis, and necroptosis; preservation of intestinal integrity; and attenuation of inflammatory responses. Consistently, AAV-mediated localized intestinal *GCLC* knockdown also attenuated the protective role of PtCuS NCs in ARIII. These findings highlight the critical role of PtCuS NCs in protecting intestinal tissues from radiation-induced injury, involving GSH-related antioxidant defenses.

Persistent intestinal damage in ARIII is driven not only by intestinal cell death but also by radiation-induced cytokine release and immune cell infiltration. Damaged intestinal epithelium and necrotic tissues release DAMPs and cytokines that recruit macrophages, which play a central role in the inflammatory cascade due to their phenotypic plasticity. Recent studies have shown that ROS-regulating nanozymes can drive macrophage phenotypic remodeling toward a pro-regenerative state and thereby promote tissue repair in inflammatory and wound-healing models [[62], [63], [64]]. In our study, PtCuS NCs downregulated pro-inflammatory cytokine levels, reduced macrophage infiltration, and modulated macrophage polarization. PtCuS NCs inhibited M1-like polarization and promoted a shift toward the M2-like phenotype. Indirectly, PtCuS NC treatment mitigated radiation-induced intestinal damage and reduced the release of inflammatory mediators, thereby downregulating the microenvironmental signals that drive M1 polarization and immune cell recruitment. This immune modulation may involve NF-κB signaling, a key regulator of macrophage activation and polarization. Suppression of NF-κB-associated M1 polarization and promotion of M2 polarization have been linked to reduced inflammation and attenuated injury-induced fibrosis [[65], [66], [67]]. Furthermore, depletion of macrophages with clodronate liposomes reduced the protective effects of PtCuS NCs on intestinal architecture and survival, indicating that macrophages are important mediators of the radioprotective effects of PtCuS NCs. These results highlight the role of PtCuS NCs in regulating macrophage infiltration and polarization to mitigate radiation-induced intestinal inflammation.

Recent studies have highlighted the role of gut microbiota dysbiosis in exacerbating ARIII. ROS induced by irradiation can facilitate the expansion of facultative anaerobic pathobionts [6]. Consistently, our data showed that irradiation disrupted gut microbiota homeostasis and increased the relative abundance of potentially pathogenic taxa, including *Enterobacteriaceae* and *Enterococcaceae*. PtCuS NC treatment markedly restored microbial diversity and enriched *Muribaculaceae* and *Prevotellaceae*, two bacterial families that have been associated with intestinal barrier maintenance and inflammatory regulation. Metabolomic analysis further showed that the altered metabolites in the PtCuS NC-treated group were mainly linked to amino acid metabolism, intestinal repair, and anti-inflammatory responses. ABX-mediated depletion of gut bacteria partially attenuated the protective effects of PtCuS NCs, whereas FMT from IR + PtCuS NC-treated donors partially alleviated ARIII-associated injury. These findings suggest that the gut microbiota contributes to PtCuS NC-mediated radioprotection, while the incomplete loss of protection after ABX treatment indicates that microbiota-independent mechanisms are also involved. Alongside the observed enhancement of intestinal GSH-related metabolism and macrophage reprogramming, these results suggest that PtCuS NCs protect against ARIII in association with the restoration of irradiation-induced microbial, metabolic, and immune disturbances.

A major concern with antioxidant-based radioprotective strategies is their potential to accelerate tumor proliferation and reduce radiotherapy efficacy. Our results indicate that PtCuS NCs do not promote tumor growth *in vitro* or *in vivo* and do not compromise the anticancer effects of radiotherapy. Increases in CD8^+^ T cell infiltration and effector GZMB ^+^ T cells were observed, suggesting that PtCuS NCs may modulate the tumor immune microenvironment. Therefore, PtCuS NCs could provide intestinal protection during radiotherapy while maintaining its antitumor efficacy, highlighting their potential as a potentially safe adjunctive strategy in tumor radiotherapy.

Our study provides preclinical evidence supporting the application of PtCuS NCs as a potential oral therapeutic candidate for ARIII. However, the single-dose 12 Gy irradiation model was chosen to induce robust intestinal injury and allow clear evaluation of the protective effects of PtCuS NCs in a controlled experimental setting. We acknowledge that clinical radiotherapy is typically delivered in fractionated doses, which may lead to different tissue responses compared with single-dose irradiation. Future studies should assess PtCuS NCs under such regimens to enhance translational relevance. In addition, although we preliminarily demonstrated favorable biocompatibility of PtCuS NCs in mouse models, the biodistribution, metabolism, long-term clearance, potential chronic toxicity, and major excretion pathways of metal nanomaterials in humans require extended and systematic evaluation in large animal models such as non-human primates. Moreover, clinical translation aspects, including scaled-up synthesis, optimization of oral formulations, and determination of dosing regimens, remain important directions for future studies. Despite these challenges, PtCuS NCs represent a promising strategy for alleviating ARIII in abdominopelvic radiotherapy.

## 5 Conclusion

**Scheme 1:**
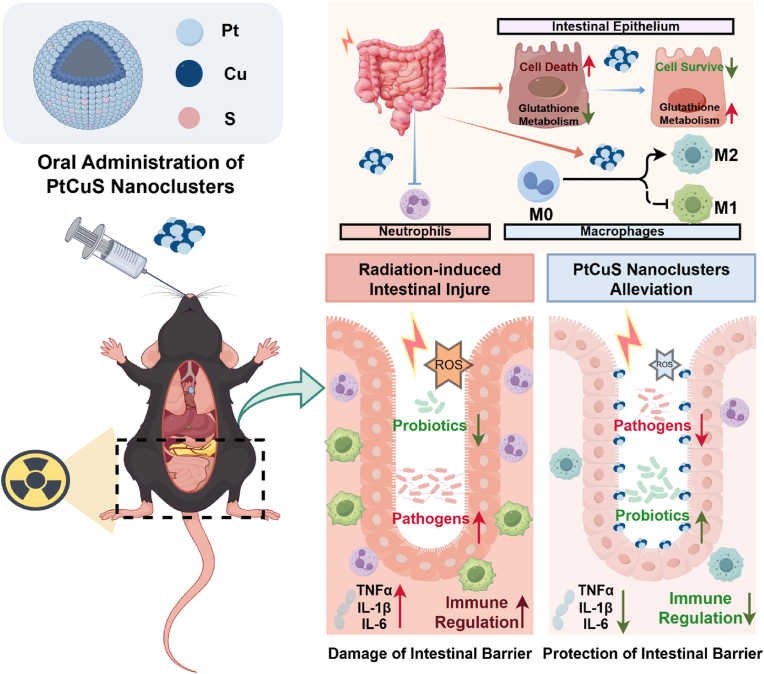
Oral PtCuS nanoclusters mitigate acute radiation-induced intestinal injury and inflammation.

We developed orally administered PtCuS NCs that effectively mitigate ARIII while maintaining radiotherapy efficacy and showing favorable biocompatibility. PtCuS NCs reduce intestinal oxidative stress and ameliorate disruptions in GSH-related metabolism after irradiation, accompanied by decreased cell death, preserved barrier integrity, and attenuated inflammatory responses. PtCuS NC treatment is also accompanied by macrophage reprogramming toward a reparative and inflammation-resolving phenotype. In addition, PtCuS NCs help restore gut microbiota homeostasis after irradiation, as reflected by increased microbial diversity, enrichment of beneficial bacteria, and suppression of the expansion of conditionally pathogenic bacteria (Scheme 1). Together, these findings suggest that PtCuS NCs confer multifaceted protection against ARIII, highlighting their clinical translation potential as a strategy to improve the quality of life of patients undergoing abdominopelvic radiotherapy.

## CRediT authorship contribution statement

**Yilin Zheng:** Conceptualization, Data curation, Investigation, Methodology, Validation, Visualization, Writing – original draft, Writing – review & editing. **Shengqi Yin:** Data curation, Formal analysis, Methodology. **Yishu Zou:** Formal analysis, Investigation, Validation. **Zehui Zhang:** Data curation, Formal analysis, Methodology, Writing – review & editing. **Junjie Li:** Data curation, Investigation, Methodology, Writing – review & editing. **Jianxin Chen:** Investigation, Validation. **Wanying Zheng:** Investigation, Validation. **Yang Liu:** Conceptualization, Methodology. **Yuqin Zhang:** Funding acquisition, Supervision. **Peiqun Yin:** Funding acquisition, Supervision, Writing – original draft, Writing – review & editing. **Yi Ding:** Conceptualization, Funding acquisition, Project administration, Supervision, Writing – review & editing.

## Declaration of competing interest

The authors declare that they have no known competing financial interests or personal relationships that could have appeared to influence the work reported in this paper.

## Data Availability

Data will be made available on request.
