# Supplementary material for: Oral of PtCuS nanoclusters mitigates acute radiation-induced intestinal injury by coordinating glutathione metabolism, macrophage repolarization, and gut microbiota
Source: Mater Today Bio. 2026 Jun 28;39:103403. doi: 10.1016/j.mtbio.2026.103403 (PMC13355488; doi:10.1016/j.mtbio.2026.103403)
Supplement: Multimedia component 1 [file mmc1.docx]

**Supplementary Information**

**Oral of PtCuS nanoclusters mitigates acute radiation-induced intestinal injury by coordinating glutathione metabolism, macrophage repolarization, and gut microbiota**

**Yilin Zheng**^a,1^, **Shengqi Yin**^a,1^, **Yishu Zou**^a,1^, **Zehui Zhang**^b,1^, **Junjie Li**^b^, **Jianxin Chen**^a^, **Wanying Zheng**^a^, **Yang Liu**^a^, **Yuqin Zhang**^a^, **Peiqun Yin**^b,*^, **Yi Ding**^a,c,**^

^a^ Department of Radiation Oncology, Nanfang Hospital, Southern Medical University, Guangzhou 510515, Guangdong Province, China.

^b^ School of Biomedical Engineering, Research and Engineering Center of Biomedical Materials, Anhui Medical University, Hefei 230032, China.

^C^ Guangdong Province Key Laboratory of Molecular Tumor Pathology, Guangzhou 510515, Guangdong Province, China.

^*^ Corresponding author. School of Biomedical Engineering, Research and Engineering Center of Biomedical Materials, Anhui Medical University, Hefei 230032, China.

^**^ Corresponding author. Department of Radiation Oncology, Nanfang Hospital, Southern Medical University, Guangzhou 510515, Guangdong Province, China.

^1^ These authors contributed equally to this work.

*E-mail addresses*: [pqyin@ustc.edu.cn](mailto:pqyin@ustc.edu.cn) (Peiqun Yin), [dy512@smu.edu.cn](mailto:dy512@smu.edu.cn) (Yi Ding).

This file includes Fig. S1-S15 and Table S1-S2

**
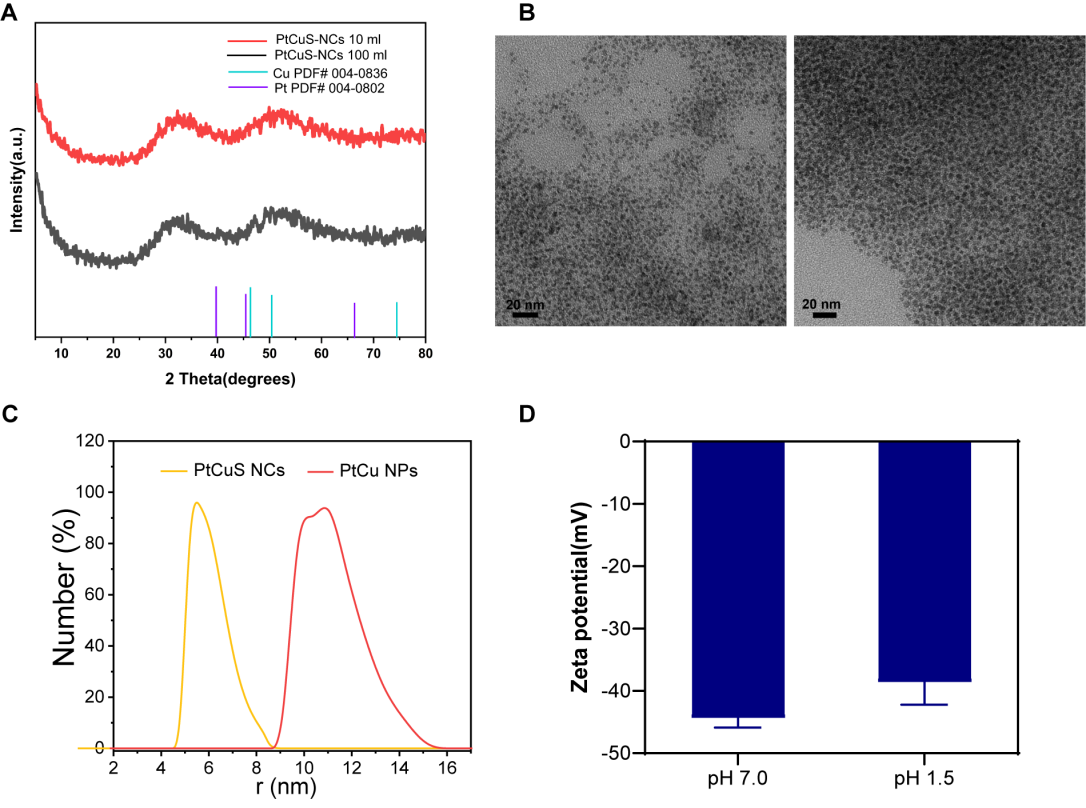
Fig. S1** Characterization of PtCuS NCs.

(A) The XRD patterns of PtCuS NCs for small vials and scale-up synthesis.

(B) The TEM images of PtCuS NCs for scale-up synthesis.

(C) Diameter distribution of PtCuS NCs and PtCu NPs.

(D) Zeta potential of PtCuS NCs in pH 7.0 and pH 1.5 buffers.


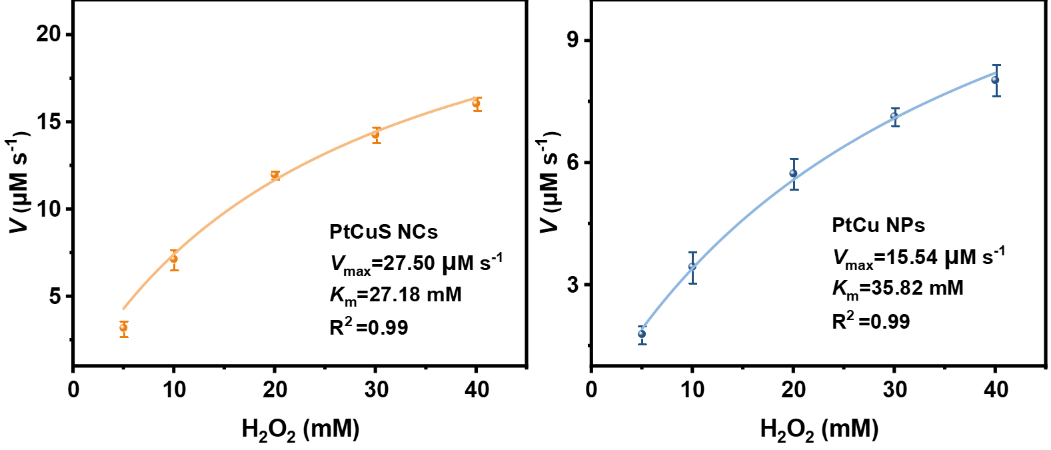


**Fig. S2** Michaelis–Menten kinetic analysis for PtCuS NCs and PtCu NPs of the CAT-mimic reaction with H_2_O_2_ substrates.

**
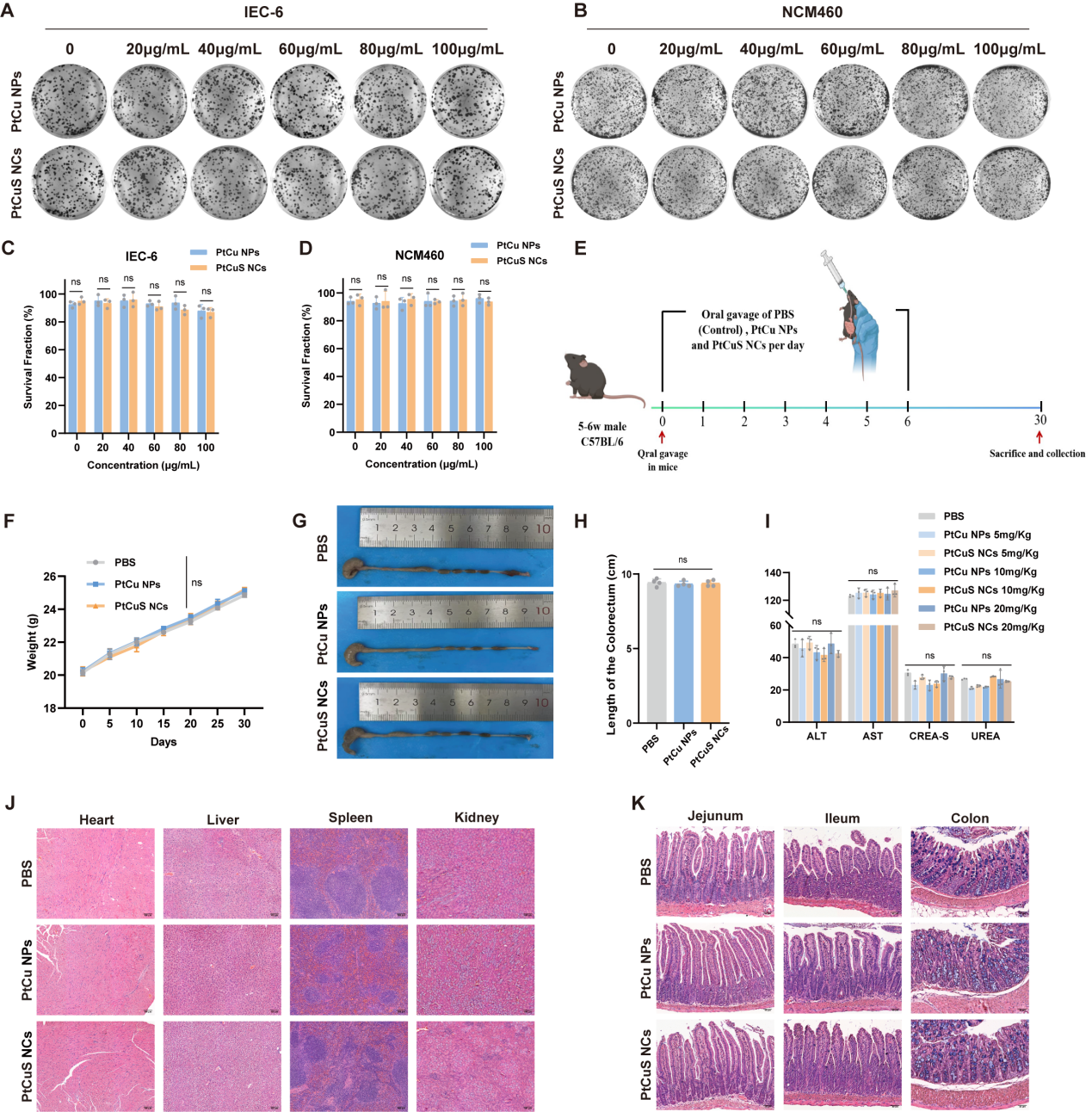
**

**Fig. S3** Biocompatibility of PtCu NPs and PtCuS NCs.

(A–D) Representative images and quantification of colony formation assays in IEC-6 and NCM460 cells treated with the indicated concentrations of PtCu NPs or PtCuS NCs. Error bars are the SD of at least three independent replicates.

(E) Schematic illustration of the C57BL/6 mouse model with oral administration of PtCu NPs and PtCuS NCs.

(F) Body weight changes of mice after oral administration of PtCu NPs or PtCuS NCs (*n*=4).

(G, H) Representative necropsy images and quantification of colorectum length in the mouse model (*n*=4).

(I) Liver and kidney function indicators in mice after oral administration of PtCu NPs or PtCuS NCs at the indicated doses (*n*=3). The units of ALT and AST are U/L. The units of CREA-S and UREA are mmol/L and μmol/L, respectively.

(J, K) Representative H&E staining of major organs and intestine. Scale bar, 40 μm.

Data were represented as mean ± SD. For *in vivo* experiments, each dot represents one biological replicate from an individual mouse. Body weight changes in panel F were analyzed using a mixed-effects model with repeated measures.Statistical significance was determined by one-way ANOVA followed by Tukey’s multiple-comparison test for panel C, D, H and I. **p* < 0.05, ***p* < 0.01, ****p* < 0.001, *****p* < 0.0001.

**
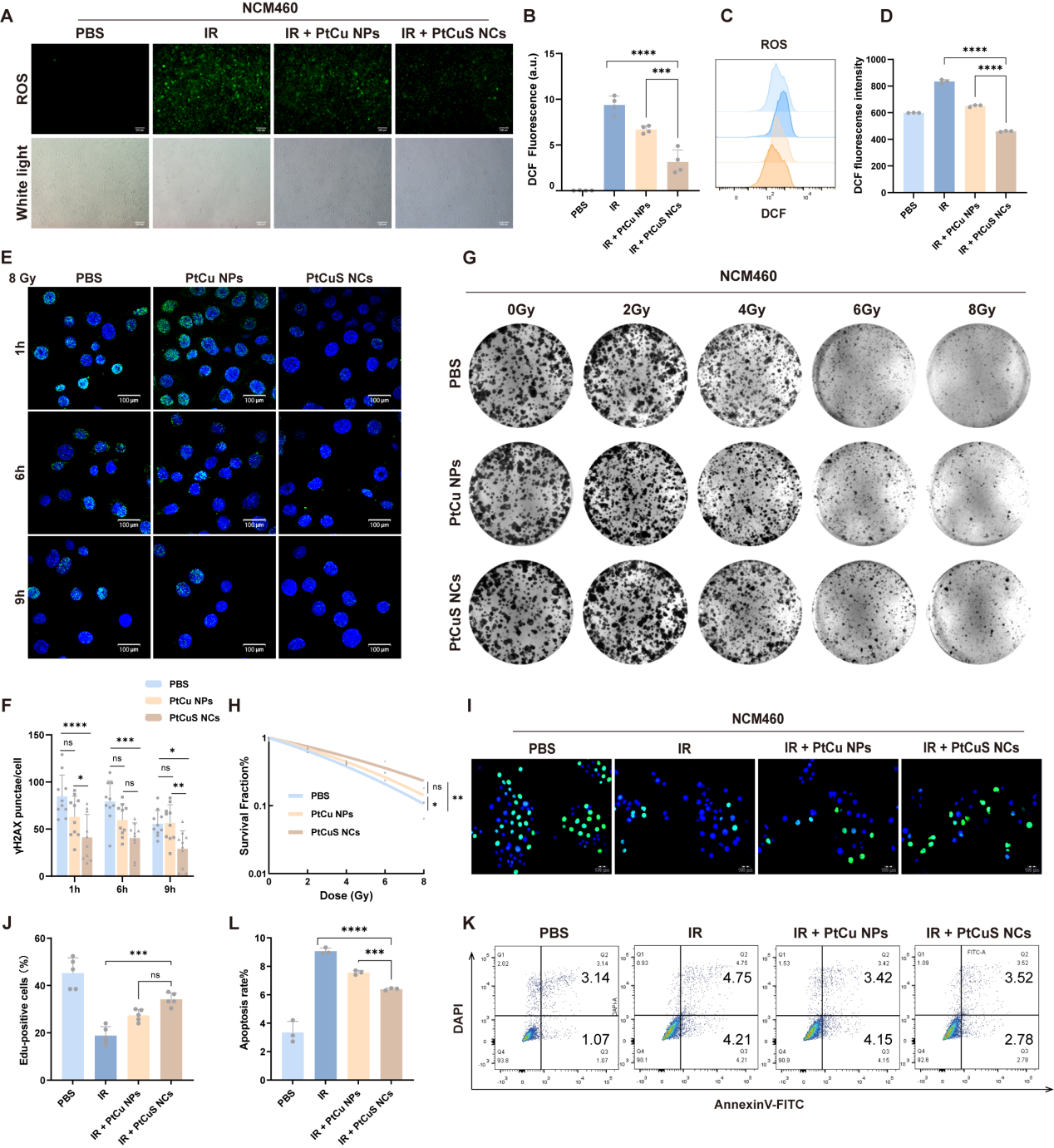
**

**Fig. S4** PtCuS NCs mitigate radiation-induced injury *in vitro.*

(A, B) Representative CLSM images and quantification of DCF staining in NCM460 cells pretreated with PtCu NPs or PtCuS NCs followed by 8 Gy irradiation (*n*=4), scale bar, 300 μm.

(C, D) Representative FCM plots and quantification of DCF fluorescence intensity in NCM460 cells (*n*=3).

(E, F) Representative images and quantification of γH2AX-Ser139 staining in NCM460 cells pretreated with PtCu NPs or PtCuS NCs followed by 8 Gy irradiation at the indicated time points. Error bars represent SD from at least ten independent replicates. Scale bar, 100 μm.

(G, H) Representative images of colony formation assays and fitted clonogenic survival curves in NCM460 cells. Error bars represent SD from at least three independent replicates.

(I, J) Representative EdU staining images and quantification in NCM460 cells under the indicated treatments (*n*=5), scale bar, 100 μm.

(K, L) Representative flow FCM plots and quantification of apoptosis in NCM460 cells under the indicated treatments following 8 Gy irradiation (*n*=3). Data are presented as mean ± SD. Statistical significance was determined by one-way ANOVA followed by Tukey’s multiple-comparison test for panels B, D, F, J, and L, and by nonlinear regression followed by the extra sum-of-squares F test for panel H. **p* < 0.05, ***p* < 0.01, ****p* < 0.001, *****p* < 0.0001.

**
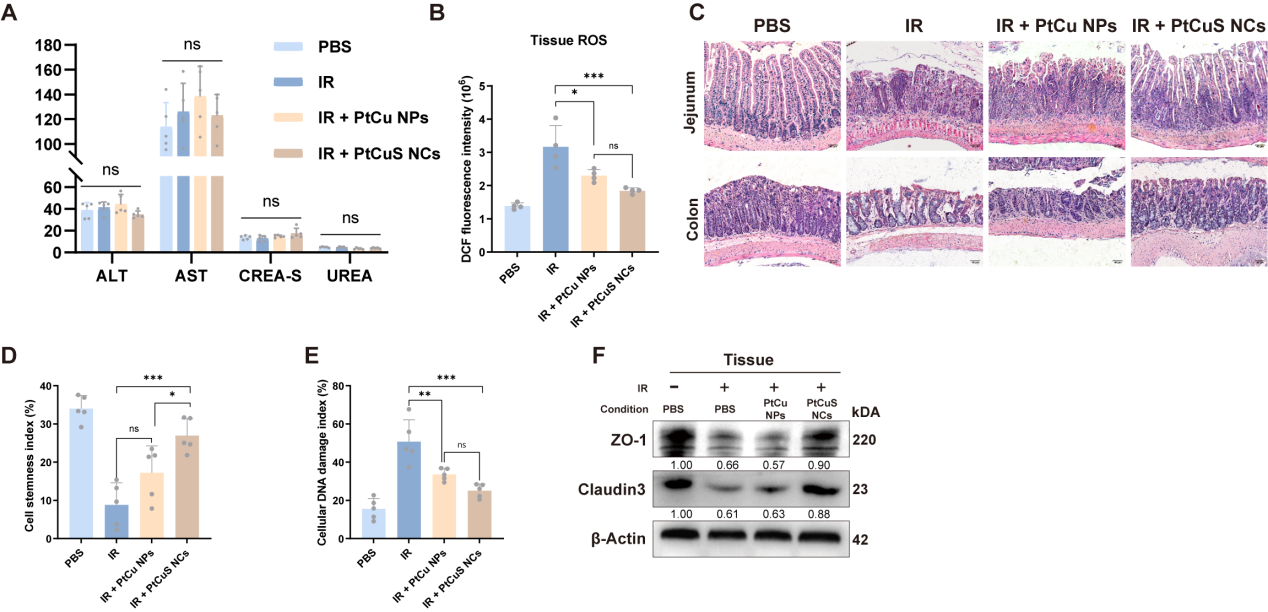
**

**Fig. S5** PtCuS NCs relieve ARIII *in vivo.*

(A) Liver and kidney function indicators in the mouse model (*n*=5). The units of ALT and AST are U/L. The units of CREA-S and UREA are Mmol/L and μmol/L, respectively.

(B) Quantification of DCF fluorescence intensity in intestinal tissue (*n*=4).

(C) Representative H&E staining images of jejunum and colon tissues from C57BL/6 mice in the indicated groups. Scale bar, 40 μm.

(D, E) Quantification of Lgr5 and γH2AX IHC staining, representing cell stemness and DNA damage, respectively, in healthy and treated mice (*n*=5). Scale bar 40 μm.

(F) Western blot analysis of intestinal barrier proteins Claudin3 and ZO-1 in indicated groups after 12 Gy irradiation *in vivo*.

Data are presented as mean ± SD. For the *in vivo* experiments, each dot represents one biological replicate from an individual mouse. In panels D and E, values for each mouse were calculated as the mean of at least three technical replicates. Statistical significance for panels A, B, D, and E was determined by one-way ANOVA followed by Tukey’s multiple-comparison test. **p* < 0.05, ***p* < 0.01, ****p* < 0.001, *****p* < 0.0001.


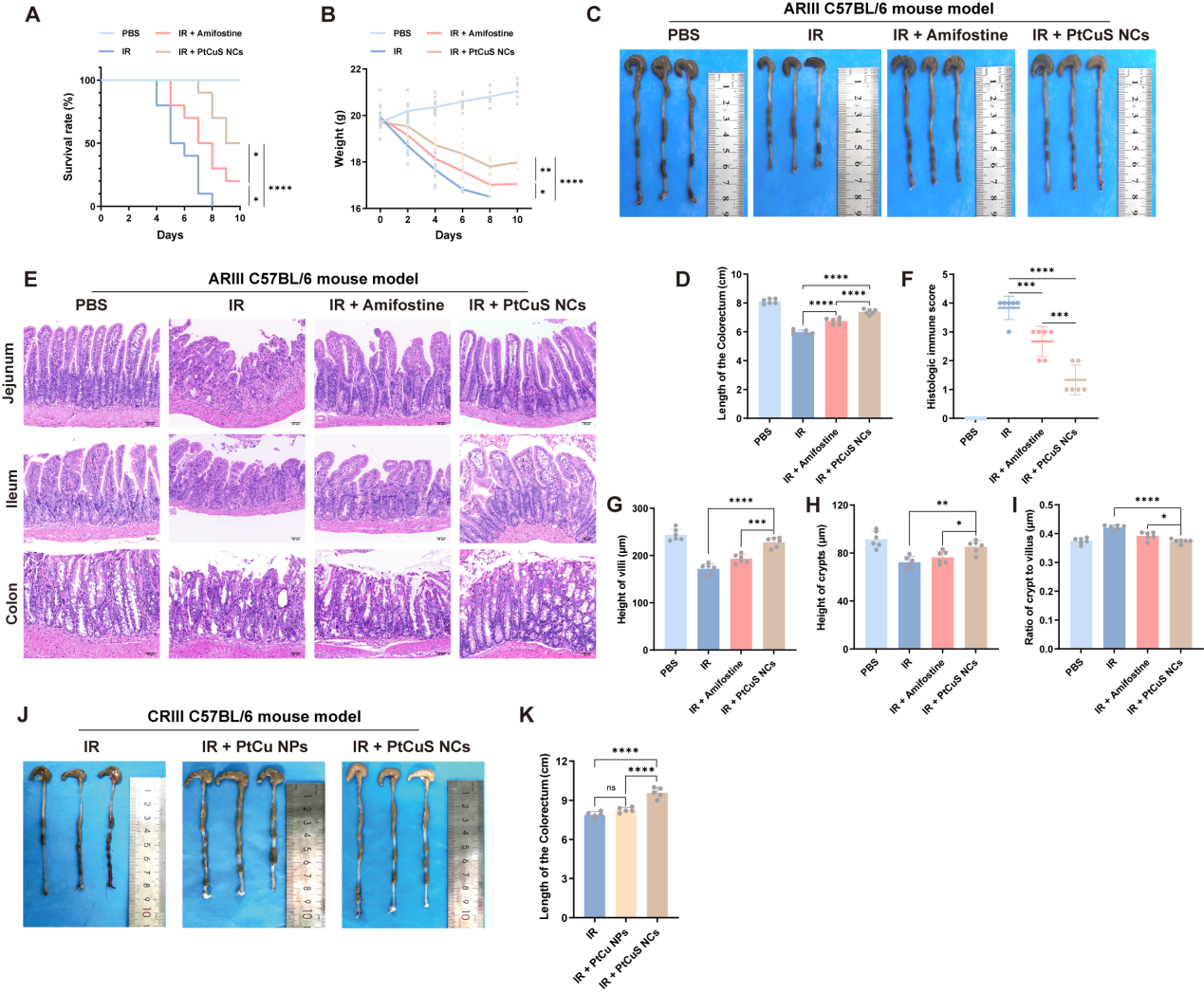


**Fig. S6** Mitigation effects of Amifostine and PtCuS NCs against ARIII and of PtCuS NCs against CRIII *in vivo.*

(A, B) Survival analysis and body weight of C57BL/6 mice in PBS, IR, IR + Amifostine and IR + PtCuS NCs groups (*n*=10).

(C, D) Representative necropsy images and quantification of colorectum length in the ARIII C57BL/6 mouse model (*n*=5).

(E) Representative H&E staining of ileal tissues from C57BL/6 mice in the indicated groups. Scale bar, 40 μm.

(F–I) Quantification of the histologic immunity scores, villus height, crypt depth and the crypt-to-villus ratio in the ileum (*n*=5).

(J, K) Representative necropsy images and quantification of colorectum length in the CRIII C57BL/6 mouse model (*n*=5).

Data are presented as mean ± SD. Each dot represents one biological replicate from an individual mouse. In panels G–I, values for each mouse were calculated as the mean of at least three technical replicates. Survival in panel A was analyzed using Kaplan–Meier curves and compared by the log-rank (Mantel–Cox) test. Body weight changes in panel B were analyzed using a mixed-effects model with repeated measures. Statistical significance for panels D, F–I, and K was determined by one-way ANOVA followed by Tukey’s multiple-comparison test. Only selected statistically relevant comparisons are shown. **p* < 0.05, ***p* < 0.01, ****p* < 0.001, *****p* < 0.0001.

**
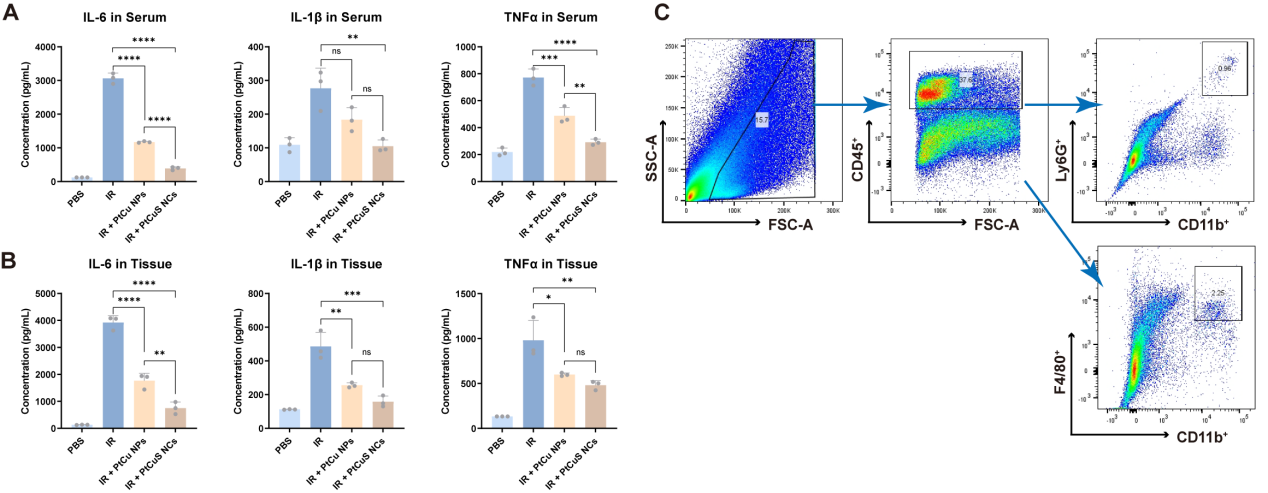
Fig. S7** PtCuS NCs relieve acute radiation-induced intestinal inflammation *in vivo.*

(A, B) ELISA analysis of IL-6, IL-1β, and TNFα levels in mouse serum and intestinal tissue, respectively (*n*=3).

(C) Gating strategies for FCM data analysis of intestinal tissue after 12 Gy irradiation *in vivo*.

Data are presented as mean ± SD. Each dot represents one biological replicate from an individual mouse. Statistical significance for panels A and B was determined by one-way ANOVA followed by Tukey’s multiple-comparison test. **p* < 0.05, ***p* < 0.01, ****p* < 0.001, *****p* < 0.0001.


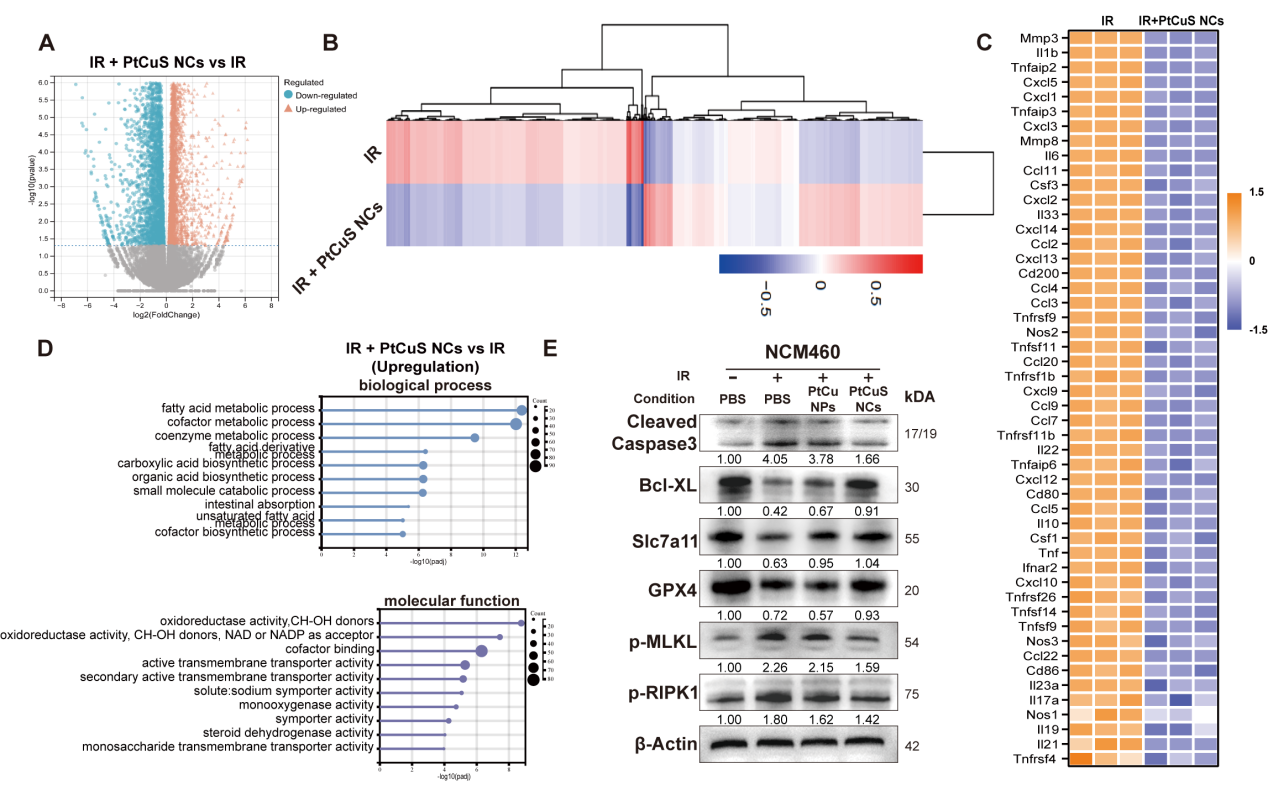


**Fig. S8** Mechanisms by which PtCuS NCs relieve ARIII.

(A) Volcano plot of differentially expressed genes in intestinal tissues from mice in the IR + PtCuS NCs and IR groups.

(B) Heatmap clustering analysis of genes in intestinal tissues from mice in the IR + PtCuS NCs and IR groups.

(C) Heatmap showing the clustering of inflammation-related genes in intestinal tissues from mice in the IR + PtCuS NCs and IR groups on day 5. Each column represents one sample (*n*=3).

(D) GO enrichment analysis of upregulated biological processes and molecular functions in intestinal tissues from the IR + PtCuS NCs group compared with the IR group.

(E) Western blot analysis of death-related protein, including Bcl-XL, cleaved Caspase-3, GPX4, Slc7A11, p-MLKL, and p-RIPK1, in the indicated groups after 8 Gy irradiation in NCM460 cells.


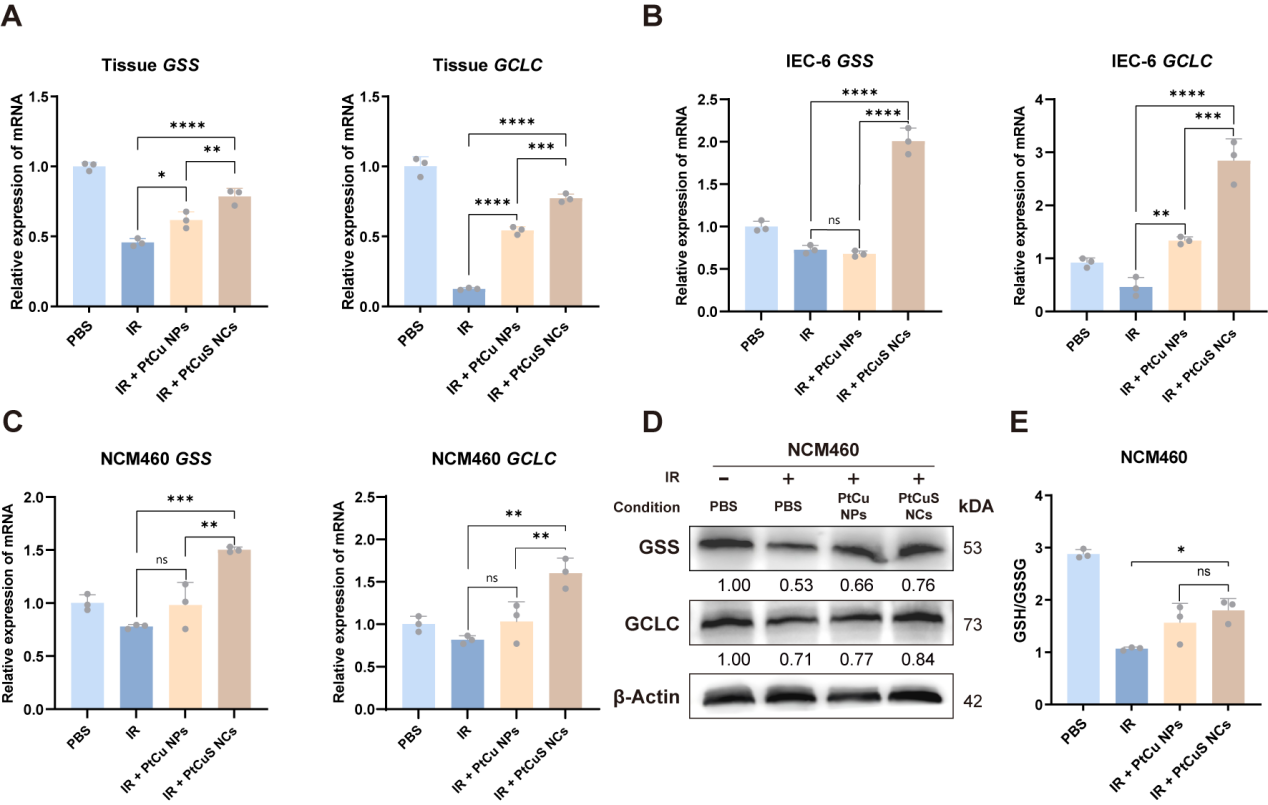


**Fig. S9** PtCuS NCs promote GSH-related metabolism after irradiation.

(A–C) Relative mRNA expression of *GSS* and *GCLC* in intestinal tissues and cells two days after irradiation (*n*=3).

(D) Western blot analysis of GSS and GCLC protein expression in NCM460 cells.

(E) Measurement of the ratio of reduced glutathione (GSH) to oxidized glutathione (GSSG) in NCM460 cells (*n*=3).

Data are presented as mean ± SD. Each dot represents one biological replicate from an individual mouse in panel A. Statistical significance for panels A–C and E was determined by one-way ANOVA followed by Tukey’s multiple-comparison test. **p* < 0.05, ***p* < 0.01, ****p* < 0.001, *****p* < 0.0001.


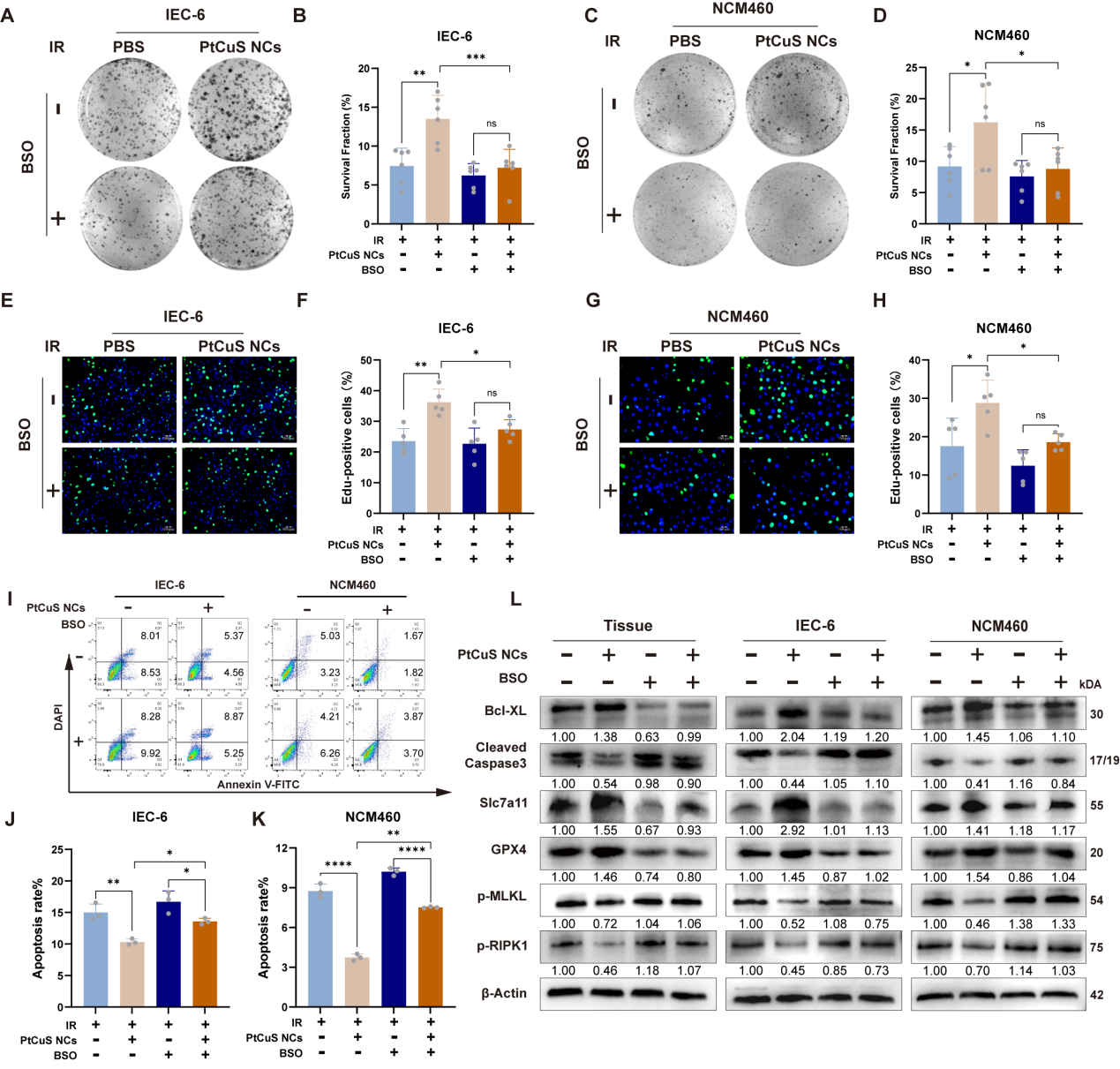


**Fig. S10** PtCuS NCs facilitate GSH metabolism pathway to alleviate radiation-induced injury *in vitro*.

(A–D) Representative images and quantification of colony formation assays in IEC-6 and NCM460 cells treated with PtCuS NCs or BSO following 8 Gy irradiation (*n*=6).

(E–H) Representative EdU staining images and quantification in IEC-6 and NCM460 cells after 8 Gy irradiation (n = 5). Scale bar, 100 μm.

(I–K) Representative FCM plots and quantification of apoptosis in IEC-6 and NCM460 cells pretreated with PtCuS NCs or BSO following 8 Gy irradiation (*n*=3).

(L) Western blot analysis of death-related protein including Bcl-XL, cleaved Caspase3, Gpx4, Slc7a11, p-MLKL and p-RIPK1 in indicated groups post 8 Gy radiation *in vitro* and *in vivo.*

Data are presented as mean ± SD. Statistical significance for panels B, D, F, H, J, and K was determined by two-way ANOVA followed by Tukey’s multiple-comparison test. **p* < 0.05, ***p* < 0.01, ****p* < 0.001, *****p* < 0.0001.


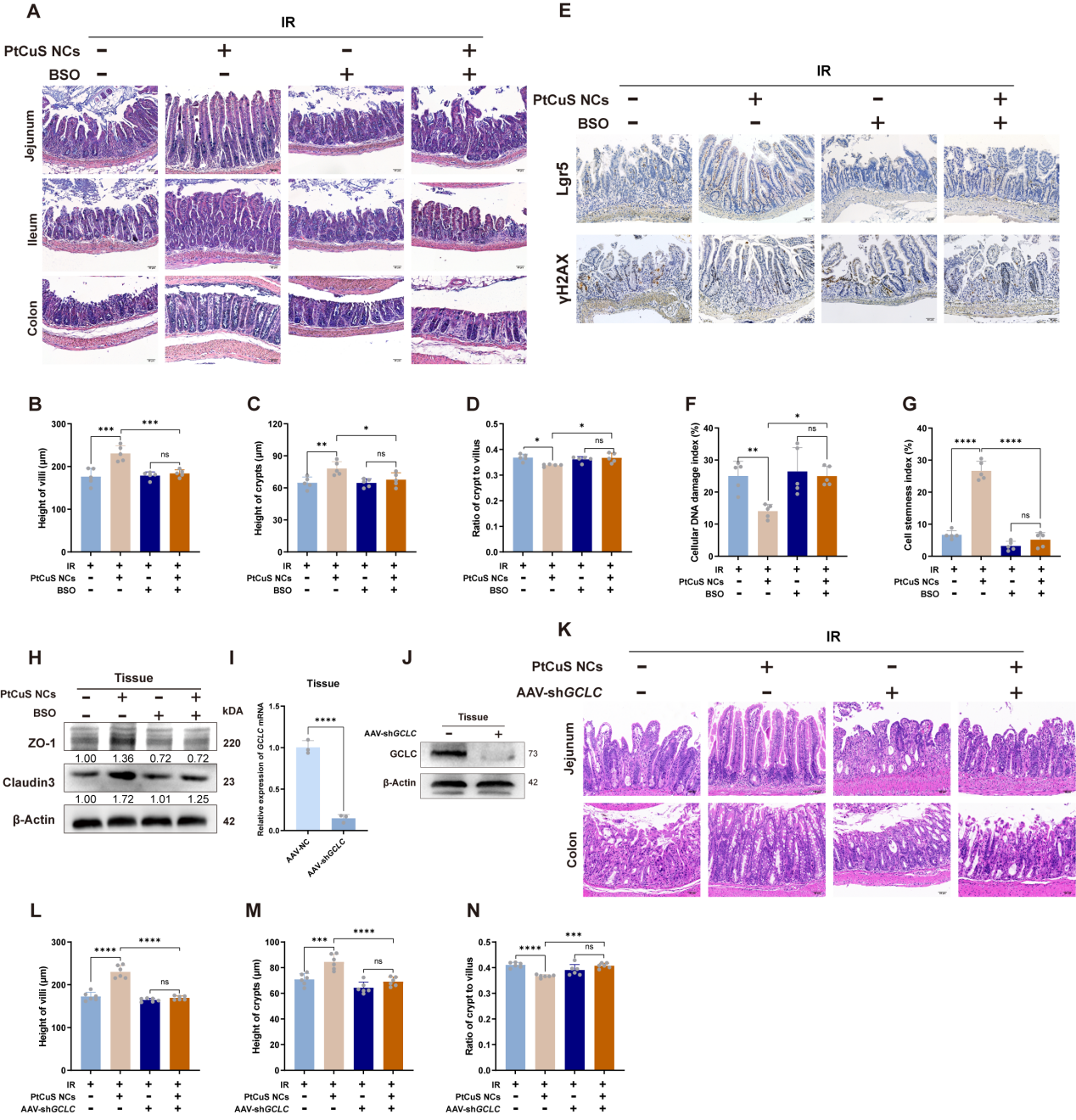


**Fig. S11** PtCuS NCs regulate GSH-related metabolism to alleviate ARIII *in vivo*.

(A) Representative H&E staining images of jejunum, ileum, and colon tissues from C57BL/6 mice treated with or without PtCuS NCs and BSO. Scale bar, 40 μm.

(B–D) Quantification of villus height, crypt depth and the crypt-to-villus ratio in the ileum from the indicated group (*n*=5).

(E–G) Representative IHC staining images and quantification of Lgr5 and γH2AX, representing cell stemness and DNA damage, respectively, in the indicated groups (*n*=5). Scale bar, 40 μm.

(H) Western blot analysis of intestinal barrier proteins Claudin3 and ZO-1 in the indicated groups following12Gy irradiation.

(I, J) qPCR and western blots of GCLC expression in the intestinal tissue of the indicated groups.

(K) Representative H&E staining images of jejunum and colon tissues from C57BL/6 mice treated with or without PtCuS NCs and AAV-sh*GCLC*. Scale bar, 40 μm.

(L–N) Quantification of villus height, crypt depth and the crypt-to-villus ratio in the ileum from the indicated group (*n*=6).

Data are presented as mean ± SD. Each dot represents one biological replicate from an individual mouse. In panels B–G and J–L, values for each mouse were calculated as the mean of at least three technical replicates. Statistical significance was determined by unpaired two-tailed Student’s t-test for panel I and by two-way ANOVA followed by Tukey’s multiple-comparison test for panels B–D, F, G, and L–N. **p* < 0.05, ***p* < 0.01, ****p* < 0.001, *****p* < 0.0001.

**
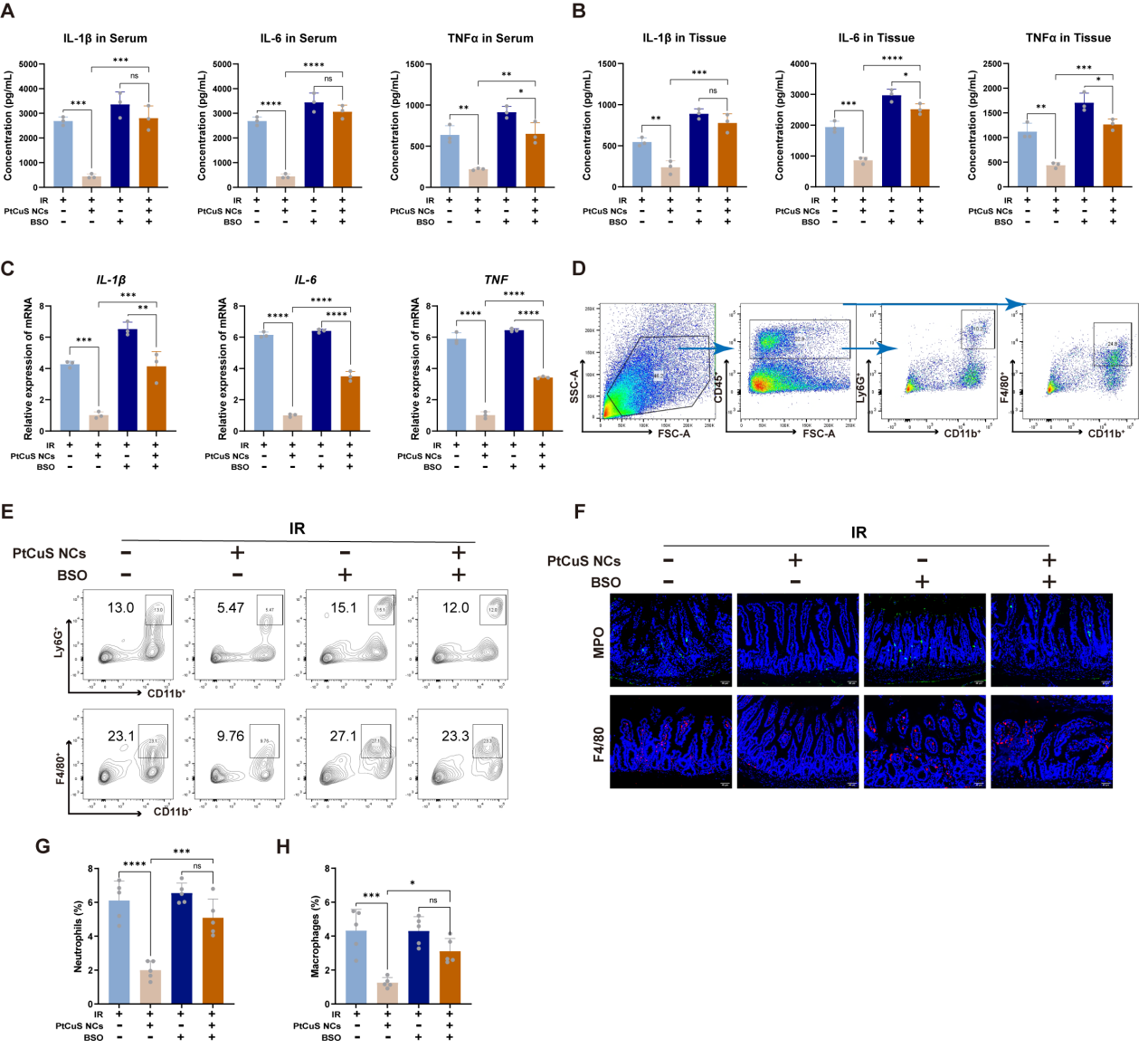
**

**Fig. S12** PtCuS NCs regulate GSH-related metabolism to alleviate radiation-induced inflammation *in vivo*.

(A, B) ELISA assays of IL-6, IL-1β, TNFα in mice serum and intestinal tissue from the indicated groups (*n*=3).

(C) qPCR analysis of *IL-6*, *IL-1β*, *TNF* in intestinal tissue from the indicated groups (*n*=3).

(D) Gating strategies for FCM analysis of intestinal tissue in the indicated groups after 12Gy irradiation *in vivo*.

(E) Representative FCM plots showing the proportion of CD11b^+^Ly6G^+^ (neutrophils) and CD11b^+^F4/80^+^ (macrophages) among CD45⁺ cells isolated from intestinal tissue in the indicated groups.

(F–H) Representative IF staining images and quantification of Ly6G^+^ (neutrophils) and F4/80^+^(macrophages) in intestinal tissue (*n*=5). Scale bar, 40 μm.

Data are presented as mean ± SD. Each dot represents one biological replicate from an individual mouse. Statistical significance for panels A–C, G, and H was determined by two-way ANOVA followed by Tukey’s multiple-comparison test. **p* < 0.05, ***p* < 0.01, ****p* < 0.001, *****p* < 0.0001.


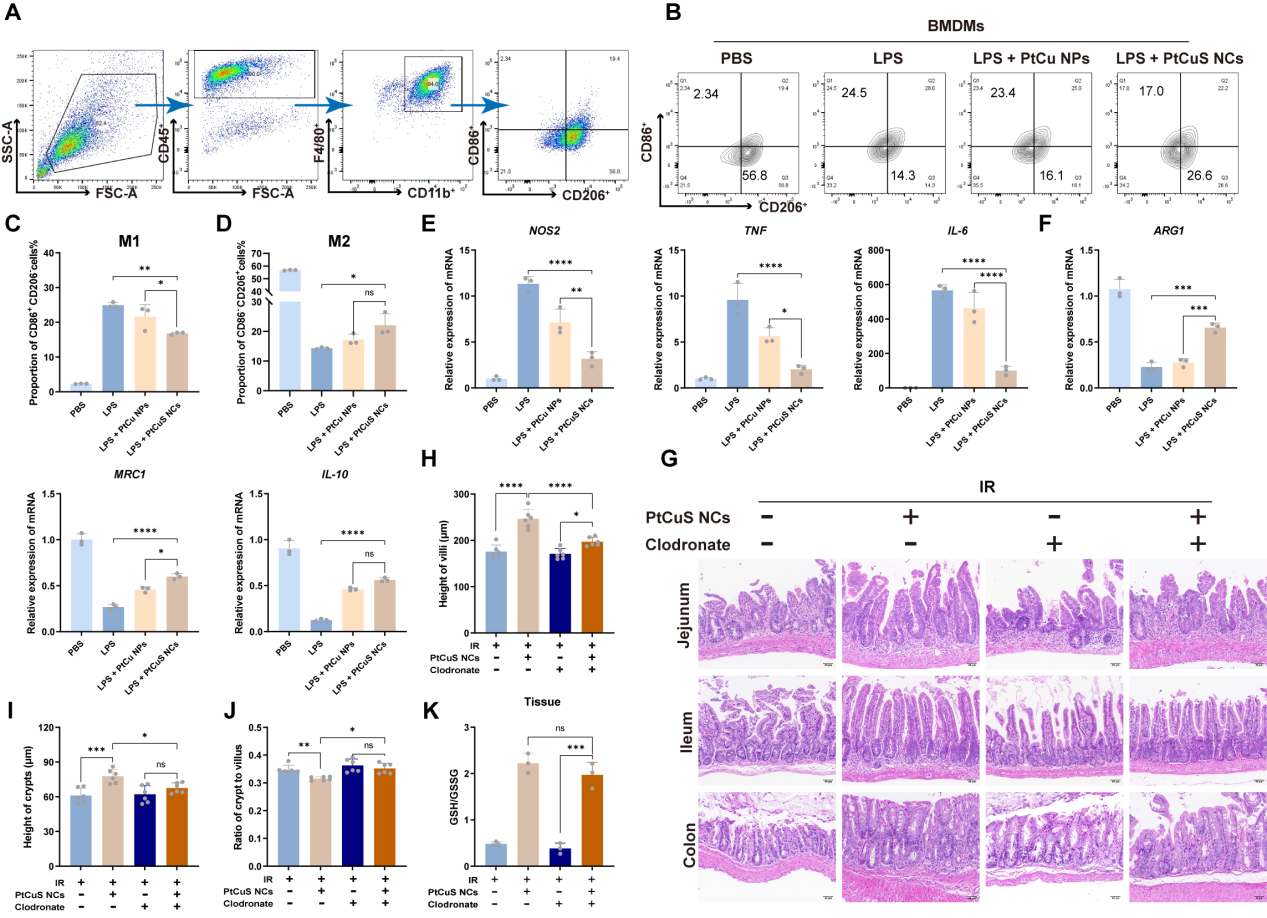


**Fig. S13** PtCuS NCs modulate macrophage polarization to mitigate ARIII.

(A) Gating strategies for FCM data analysis of CD86 and CD206 in indicated groups.

(B–D) Representative FCM plots and quantification of the proportions of M1-like CD86⁺CD206⁻ and M2-like CD86⁻CD206⁺ BMDMs in the indicated groups (*n*=3).

(E, F) qPCR of M1 markers (*NOS2*, *TNF* and *IL-6*) and M2 markers (*ARG1*, *MRC1* and *IL-10*) from BMDMs in indicated groups (*n*=3).

(G) Representative H&E staining images of jejunum, ileum, and colon tissues from C57BL/6 mice in the indicated groups. Scale bar, 40 μm.

(H–J) Quantification of villus height, crypt depth and the crypt-to-villus ratio in the ileum from indicated group (*n*=6).

(K) Measurement of the ratio of reduced glutathione (GSH) to oxidized glutathione (GSSG) in the indicated mouse groups (*n*=3).

Data are presented as mean ± SD. For the *in vivo* experiments, each dot represents one biological replicate from an individual mouse. In panels H–K, values for each mouse were calculated as the mean of at least three technical replicates.Statistical significance was determined by one-way ANOVA followed by Tukey’s multiple-comparison test for panels C–F and by two-way ANOVA followed by Tukey’s multiple-comparison test for panels H–K. **p* < 0.05, ***p* < 0.01, ****p* < 0.001, *****p* < 0.0001.


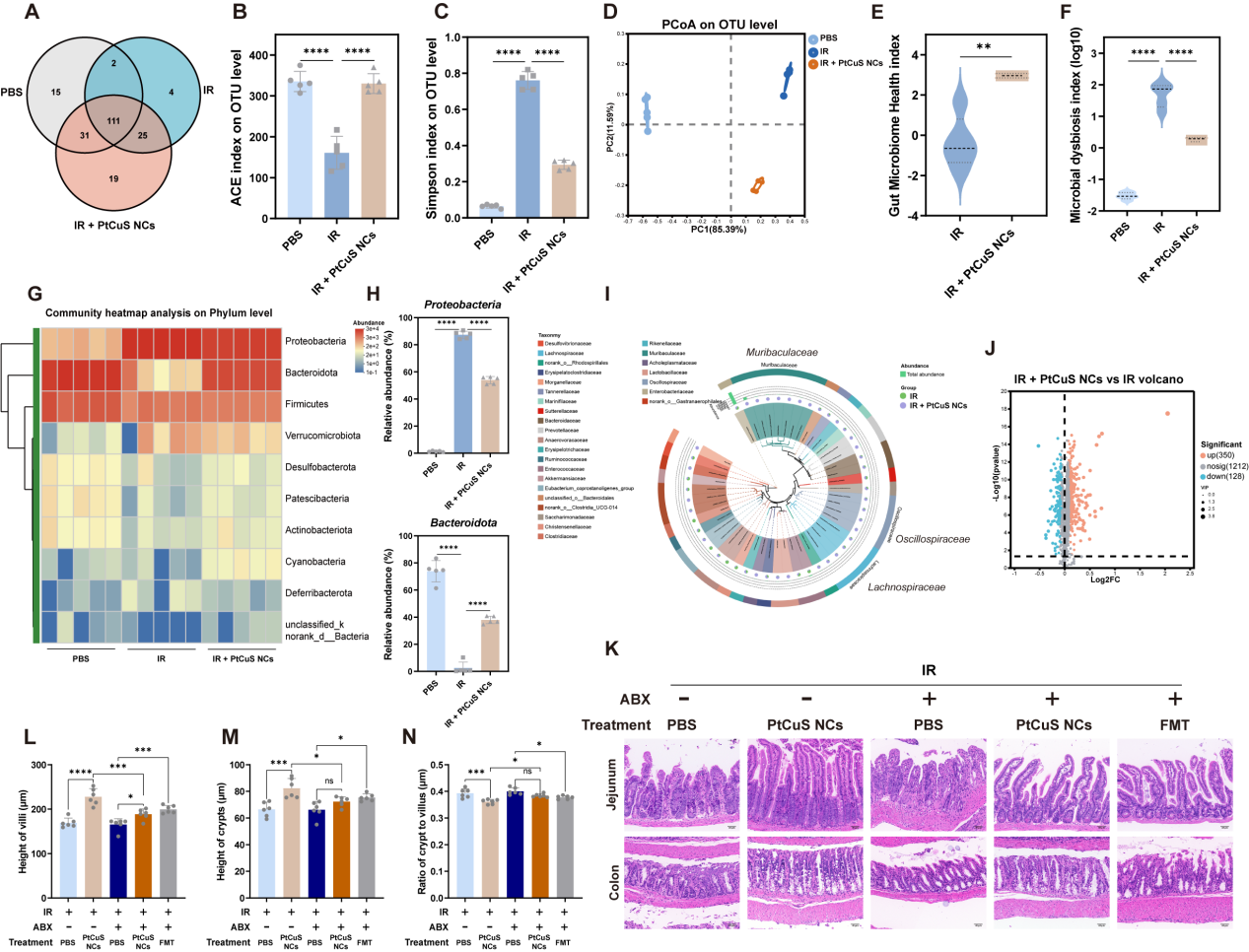


**Fig. S14** PtCuS NCs attenuate gut microbiota dysbiosis and modulate metabolites.

(A) Venn diagram of the gut microbiota composition among groups on the operational taxonomic units (OTUs) level.

(B, C) ACE and Simpson indices of fecal microbiota in the indicated groups (*n*=5)*.*

(D) PCoA analysis of fecal microbiota in the indicated groups (*n*=5)*.*

(E, F) Microbial Dysbiosis Index (MDI) and Gut Microbiome Health Index (GMHI) of fecal microbiota in the indicated groups (*n*=5).

(G) Taxonomic composition analysis at the phylum level.

(H) Relative abundance of *Proteobacteria* and *Bacteroidota* in fecal microbiota from the indicated groups (*n*=5).

(I) Circular phylogenetic tree showing taxonomic ranks from phylum to species from the inside to the outside.

(J) Volcano plot of metabolite composition between the IR + PtCuS NCs and IR groups.

(K) Representative H&E staining images of jejunum, ileum, and colon tissues from C57BL/6 mice in the indicated groups. Scale bar, 40 μm.

(L–N) Quantification of villus height, crypt depth and the crypt-to-villus ratio in the ileum from the indicated group (*n*=6).

Data are presented as mean ± SD. Each dot represents one biological replicate from an individual mouse. In panels L–N, values for each mouse were calculated as the mean of at least three technical replicates. Statistical significance was determined by Welch’s t-test for panel E and by one-way ANOVA followed by Tukey’s multiple-comparison test for panels B, C, F, and H. For panels L–N, two-way ANOVA followed by Tukey’s multiple-comparison test was used for comparisons among the IR, IR + PtCuS NCs, IR + ABX, and IR + ABX + PtCuS NCs groups, whereas one-way ANOVA followed by Tukey’s multiple-comparison test was used for comparisons among the IR, IR + ABX, and IR + ABX + FMT groups. **p* < 0.05, ***p* < 0.01, ****p* < 0.001, *****p* < 0.0001.


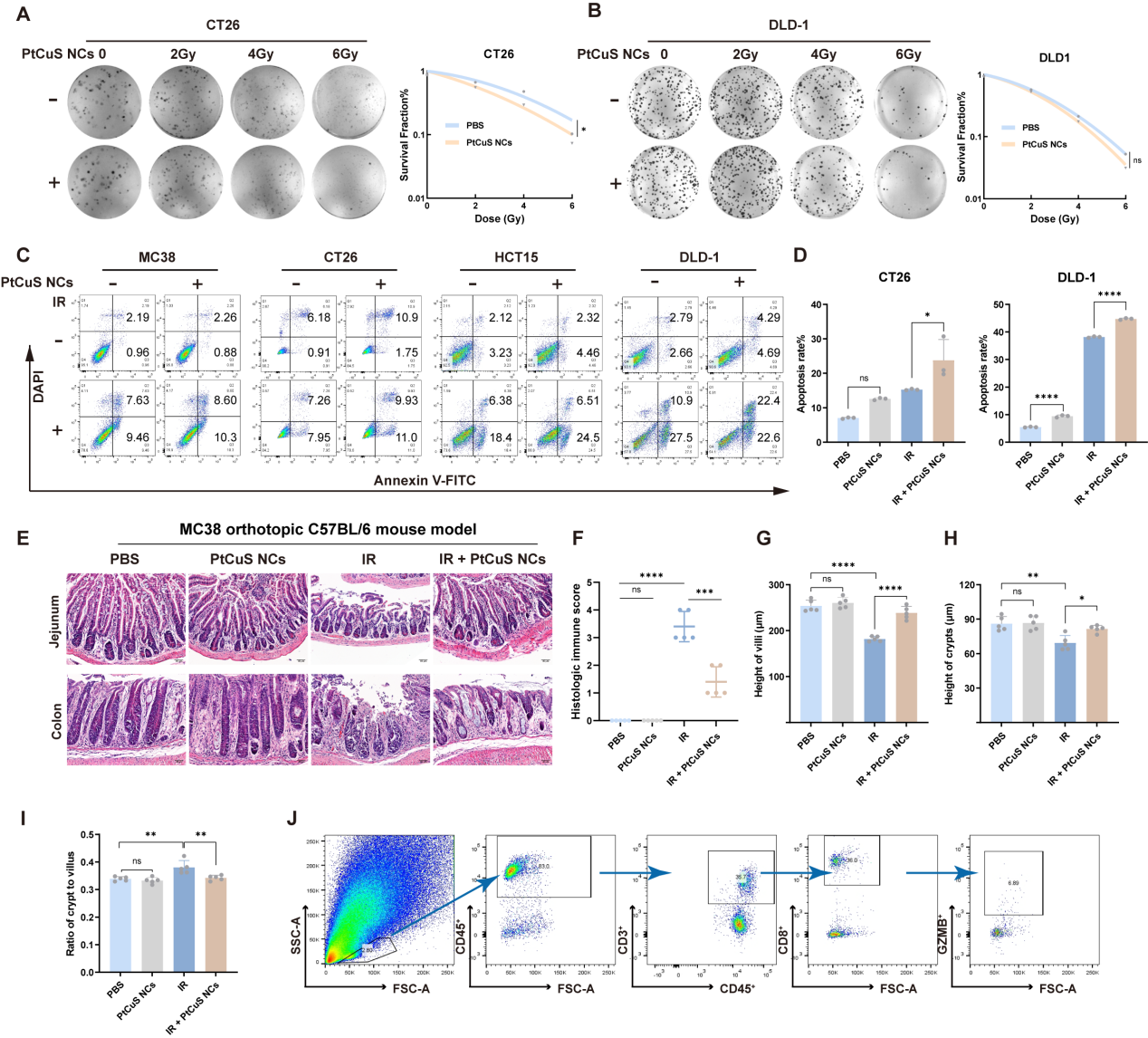


**Fig. S15** PtCuS NCs do not compromise the efficacy of radiotherapy.

(A, B) Representative images of colony formation assays and fitted clonogenic survival curves in CT26 and DLD-1 cells treated with or without PtCuS NCs following irradiation at the indicate (*n*=3).

(C) Representative FCM plots of apoptosis in MC38, CT26, HCT15, and DLD-1 cells treated with or without PtCuS NCs following 8 Gy irradiation.

(D) Quantification of apoptosis in CT26 and DLD-1 cells treated with or without PtCuS NCs following 8 Gy irradiation (*n*=3).

(E) Representative H&E staining images of jejunal tissues from MC38 orthotopic tumor-bearing mice. Scale bar, 40 μm.

(F) Quantification of histological injury scores (*n*=5).

(G–I) Quantification of villus height, crypt depth and the crypt-to-villus ratio in the ileum from the indicated group (*n*=5).

(J) Gating strategies for FCM analysis of tumor tissue from MC38 orthotopic tumor-bearing mice treated with or without PtCuS NCs after 12 Gy irradiation.

Data are presented as mean ± SD. For the *in vivo* experiments, each dot represents one biological replicate from an individual mouse. In panels G–I, values for each mouse were calculated as the mean of at least three technical replicates. Statistical significance for panels A and B was determined by nonlinear regression followed by the extra sum-of-squares F test. Statistical significance for panels D and F–I was determined by two-way ANOVA followed by Tukey’s multiple-comparison test.**p* < 0.05, ***p* < 0.01, ****p* < 0.001, *****p* < 0.0001.

Table. S1: Comparison of CAT-mimic kinetic parameters of PtCuS NCs with other Pt or Cu-based nanozymes.

| **Nanozymes** | ***V_max_* (μM s^-1^)** | ***K_m_* (mM)** | **pH** | **References** |
| --- | --- | --- | --- | --- |
| **Pt-INOS@ZIF** | 3.46 | 172 | 7.4 | [1] |
| **PtsaN-C** | 2.78 | 19.33 | 7.4 | [2] |
| **AuPtCo** | 0.10 | 2.593 | 4.0 | [3] |
| **CuN_3_-Sazyme** | 1.46 | 65.8 | 3.5 | [4] |
| **MCCP** | 4.75 | 2.02 | 5.0 | [5] |
| **Pt@CNDs** | 0.04 | 42.84 | 7.4 | [6] |
| **PtCu NPs** | 15.54 | 35.82 | 7.4 | This work |
| **PtCuS NCs** | 27.5 | 27.18 | 7.4 | This work |

Table S2: ICP-MS quantification of PtCuS NCs in the mouse intestinal tract at 1, 4, 8, and 12 h post-oral administration, expressed as wt% of total tissue. All mice received equivalent initial Pt doses to ensure consistent comparison.

| **Time (h)**  **Sample** | **PtCu NPs** | **PtCuS NCs** |  |
| --- | --- | --- | --- |
| **1** | 5.7 | 7.0 | **Pt (10^-3^ wt%)** |
| **4** | 3.5 | 6.6 |  |
| **8** | 2.6 | 4.1 |  |
| **12** | 1.8 | 2.6 |  |

**References:**

[1] J. Mu, C. Li, Y. Shi, G. Liu, J. Zou, D. Zhang, C. Jiang, X. Wang, L. He, P. Huang, Y. Yin, X. Chen, Protective effect of platinum nano-antioxidant and nitric oxide against hepatic ischemia-reperfusion injury, NAT COMMUN, 13 (2022) 2513.

[2] T. Ye, C. Chen, D. Wang, C. Huang, Z. Yan, Y. Chen, X. Jin, X. Wang, X. Ding, C. Shen, Protective effects of Pt-N-C single-atom nanozymes against myocardial ischemia-reperfusion injury, NAT COMMUN, 15 (2024) 1682.

[3] X. Zhou, C. Fan, Q. Tian, C. Han, Z. Yin, Z. Dong, S. Bi, Trimetallic AuPtCo Nanopolyhedrons with Peroxidase- and Catalase-Like Catalytic Activity for Glow-Type Chemiluminescence Bioanalysis, ANAL CHEM, 94 (2022) 847-855.

[4] J. Wu, X. Zhu, Q. Li, Q. Fu, B. Wang, B. Li, S. Wang, Q. Chang, H. Xiang, C. Ye, Q. Li, L. Huang, Y. Liang, D. Wang, Y. Zhao, Y. Li, Enhancing radiation-resistance and peroxidase-like activity of single-atom copper nanozyme via local coordination manipulation, NAT COMMUN, 15 (2024) 6174.

[5] J. Zhou, D. Xu, G. Tian, Q. He, X. Zhang, J. Liao, L. Mei, L. Chen, L. Gao, L. Zhao, G. Yang, W. Yin, G. Nie, Y. Zhao, Coordination-Driven Self-Assembly Strategy-Activated Cu Single-Atom Nanozymes for Catalytic Tumor-Specific Therapy, J AM CHEM SOC, (2023) 10.1021/jacs.2c13597.

[6] Y.J. Zhang, W.H. Gao, Y.A. Ma, L.L. Cheng, L. Zhang, Q.G. Liu, J.Y. Chen, Y.R. Zhao, K.S. Tu, M.Z. Zhang, C. Liu, Integrating Pt nanoparticles with carbon nanodots to achieve robust cascade superoxide dismutase-catalase nanozyme for antioxidant therapy, NANO TODAY, 49 (2023).
